# Adaptation is influenced by the complexity of environmental change during evolution in a dynamic environment

**DOI:** 10.1371/journal.pgen.1009314

**Published:** 2021-01-25

**Authors:** Sébastien Boyer, Lucas Hérissant, Gavin Sherlock

**Affiliations:** Department of Genetics, Stanford University, Stanford, California, United States of America; University College Dublin, IRELAND

## Abstract

The environmental conditions of microorganisms’ habitats may fluctuate in unpredictable ways, such as changes in temperature, carbon source, pH, and salinity to name a few. Environmental heterogeneity presents a challenge to microorganisms, as they have to adapt not only to be fit under a specific condition, but they must also be robust across many conditions *and be able to deal with the switch between conditions itself*. While experimental evolution has been used to gain insight into the adaptive process, this has largely been in either unvarying or consistently varying conditions. In cases where changing environments have been investigated, relatively little is known about how such environments influence the dynamics of the adaptive process itself, as well as the genetic and phenotypic outcomes. We designed a systematic series of evolution experiments where we used two growth conditions that have differing timescales of adaptation and varied the rate of switching between them. We used lineage tracking to follow adaptation, and whole genome sequenced adaptive clones from each of the experiments. We find that both the switch rate and the order of the conditions influences adaptation. We also find different adaptive outcomes, at both the genetic and phenotypic levels, even when populations spent the same amount of total time in the two different conditions, but the order and/or switch rate differed. Thus, in a variable environment adaptation depends not only on the nature of the conditions and phenotypes under selection, but also on the complexity of the manner in which those conditions are combined to result in a given dynamic environment.

## Author summary

The environments in which organisms evolve typically fluctuate, in both predictable ways, such as daily, seasonal and annual changes in, for example, temperature and photoperiod, as well as in hard to predict ways, such as changes in the weather. Most laboratory evolution experiments evolve organisms in either constant environments, or environments that change predictably, such as by serial dilution into fresh media periodically. To investigate how unpredictable changes in the environment can affect an organism’s evolution, we designed a series of experiments where the environment alternated between two conditions, either predictably, or randomly, with different timescales of switching. We then evolved the budding yeast, *Saccharomyces cerevisiae* under these different conditions. We found that the both the switch rate and the order of the conditions influences adaptation, and that switching can both speed up and slow down adaptation. We also observed different adaptive outcomes between populations, both at the genetic and phenotypic level, even if populations spent the same amount of total time in the two different conditions, but that the order and/or switch rate differed. These data suggest that adaptive outcomes are dependent on the exact nature of the prevailing environmental conditions.

## Introduction

How organisms evolve is a fundamental question in biology, and how they adaptively evolve in response to *changing* environments is a question whose answer is central to rational vaccine development [1], as well as to understanding the evolution of multiple antibiotic resistance [2,3], the evolution of immune systems [4], and even heritability [5]. In nature, some environmental changes are predictable, and organisms can evolve responses to such predictable changes. For example, *E*. *coli* shows asymmetric anticipation of carbon sources, such that in the presence of lactose, *E*. *coli* anticipates that maltose will soon become available, because this is what has been repeatedly experienced in the mammalian gut. Mechanistically, this is due to lactose modestly inducing the genes required for maltose metabolism [6]. However, when wild *E*. *coli* are grown under laboratory conditions, which typically lack this selective pressure, this “anticipation” is lost as the strain undergoes domestication [6]. Likewise, circadian clocks are thought to provide a fitness benefit, allowing organisms to adapt physiologically to diurnal changes in light, temperature, and humidity [7]. In Cyanobacteria, the benefit of a circadian clock can only be maintained in the lab by continued exposure to a rhythmic environment [8]. Environmental change may vary based on the frequency of switching, and whether the switching is random or predictable–one way in which organisms can adapt to deal with environmental uncertainty is by bet hedging, whereby by the stochastic switching between different phenotypic states can allow a portion of a population to be more fit under a certain environment[9]. It has been experimentally shown that bet-hedging approaches that resulting in greater average fitness across environments can be engineered [10] or evolved [11,12], and there is a rich theory on bet hedging as a strategy to survive in variable environments that switch more rapidly than can be kept up with through mutation and selection alone [13,14].

Experimental Microbial Evolution (EME [15]; also referred to as Adaptive Laboratory Evolution (ALE)) is a prospective approach to studying adaptive evolution in the laboratory and was first used ~140 years ago [16]. EME has been used to address fundamental evolutionary questions, such as the rate at which beneficial mutations fix [17], and the influence of both ploidy [17] and sex [18] on that rate. High-throughput sequencing has made it possible to establish at high resolution how mutations accumulate in co-evolving lineages, revealing clonal interference, with hundreds or thousands of beneficial lineages competing [19–22], sometimes even with multiple lineages persisting in a quasi-stable state for thousands of generations [23]. While EME has provided many insights into the evolutionary process (see [24,25] for reviews), such experiments have typically been performed in either constant environments (such as the chemostat), consistently fluctuating environments (as in by serial transfer), or in environments where a variable of interest changes monotonically over either time, as in a morbidistat[26], or over space, as in the mega-plate experiment [27]. However, outside of the laboratory organisms are almost never challenged to adaptively evolve in such predictable environments, but rather must cope with variability and stochasticity. To date, only a few EME studies (see [28] for review) have sought to determine either how microbes adapt to unpredictable changes in the environment, or what characteristics of such changes might be important in influencing adaptation. For example, when *Pseudomonas fluorescens* was evolved in variable environments, switching between contrasting carbon sources (xylose and mannose), it was found, contrary to expectation, that populations frequently evolved to be niche specialists, and became adapted to the less favorable carbon source [29]. By contrast, when evolving in a heterogeneous environment containing multiple carbon sources, adaptation converged on the most productive carbon source [30]. In another example, a recent study investigated the fitness of the yeast deletion collection under different time scales of periodic environmental change and showed that some mutants are better at dealing with the environmental switch itself, suggesting that it is possible to evolve genotypes that are adapted to change, *per se* [31]. To date no study has characterized the dynamics of evolution during adaptation to a changing environment or asked specifically how these dynamics might change as a function of the switch rate and strength of selection.

To fill this gap, and to improve our knowledge of how dynamic environments impact the evolutionary process, a systematic (for a given set of environments) exploration of the parameters of dynamic environments is needed, to determine how these parameters affect evolutionary dynamics, and the fitness effects of adaptive mutations across environments. Here we present a series of experiments that explore evolution during switching between two environmental conditions (glucose-containing medium with fluconazole, vs. medium containing ethanol/glycerol with no drug), varying two important parameters: 1) the degree of randomness of the switches between the two conditions, and 2) the consecutive time spent in each condition. Using DNA barcode-based lineage tracking we followed the evolutionary dynamics in 8 different environmental scenarios, investigating the statistics of the evolutionary dynamics, and determining the phenotypic and genotypic characteristics of adaptive mutants arising in each. We found that the speed of adaptation is influenced by the rate of switching between conditions, and that different switching dynamics could lead to the selection of clones with very different behaviors in each environment. Finally, we found that different environmental sequences select for different phenotypic and genotypic outcomes; for example, a randomly switching environment tended to select for generalists, while a consistent strong selection in a non-switching environment selected for specialists.

## Results

### Experimental design and overview

**Fig 1:**
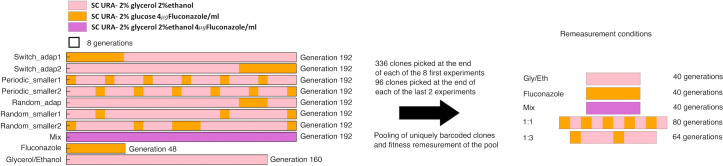
Experimental design. Each experiment with a fluctuating environment was constructed using blocks of 8 generations in Fluconazole and 24 generations in Gly/Eth. At the end of 192 generations, the total time spent in Fluconazole is 48 generations and 144 generation in Gly/Eth, for switch_adap1, switch_adap2, periodic_smaller1, periodic_smaller2 and random_smaller2 experiments. By contrast, in random_adap1 and random_smaller1 the total time spent in Fluconazole is 24 generations, with 168 generations in Gly/Eth. Two additional experiments, which did not switch between environments, were also carried out. Clones isolated from each experiment were pooled, and then had their fitness remeasured under 5 different conditions (right).

**Fig 2:**
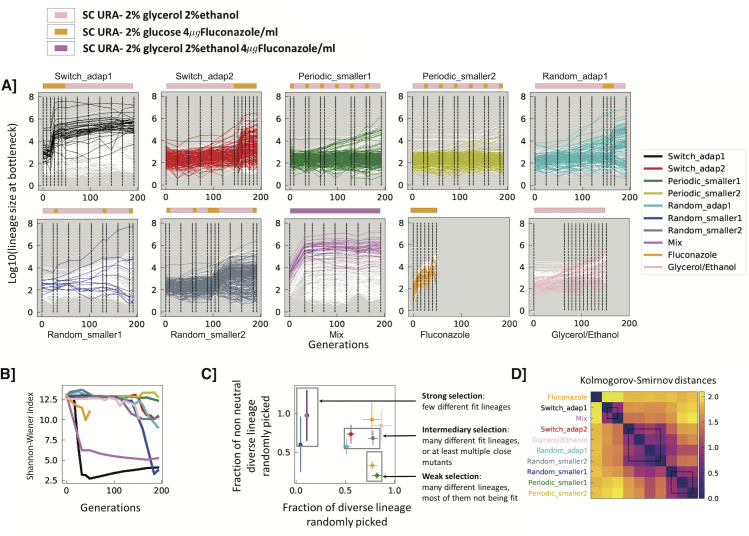
**A] Lineage tracking data for a subsample of 300 lineages for each experiment over the course of 192 generations**. Colored lines correspond to lineages for which single colonies were later isolated; white lines correspond to a randomly selected set lineages which were not sampled for fitness remeasurement. Dashed lines represent sampled time points. Environments are indicated by the color strips above each graph, with colors as in Fig 1. **B] Shannon-Wiener index, calculated using frequencies for all lineages in each experiment. C] Strength of selection.** 336 lineages were randomly picked from each experiment at generation 192, and we determined the fraction that were non-neutral (y-axis) and how many unique barcodes there were, out of 336 (x-axis). Error bars indicate error based on counting statistics. D] **Similarity of experimental conditions**. Kolmogorov Smirnov distances were calculated between all the experimental conditions based on the fitness remeasurement data (see Methods for details). The distance matrix was hierarchically clustered.

We evolved, by serial transfer, barcoded diploid yeast populations in dynamic environments built using two single environment blocks (see Fig 1 for experimental design), varying two main parameters: i) the time spent in each particular environment, relative to the timescale of adaptation within that environment and ii) the periodicity/randomness of the switching between environments. We defined the timescale of adaptation as the evolutionary time required for a certain fraction of the population to be adaptive within a given environment: for diploid yeast evolving by serial transfer in synthetic complete (SC) medium with 2% glucose + 4 μg/ml Fluconazole (hereafter referred to as “Fluconazole”), ~20% of the population is adaptive after 48 generations, while, in SC medium with 2% glycerol and 2% ethanol (“Gly/Eth”), the timescale of adaptation is much longer: ~15% of the population is adaptive after 144 generations (Fig 2A and Humphrey, Hérissant et al, in prep.). The timescale of adaptation for these two environments is thus 48 and 144 generations respectively. We designed 8 different evolution experiments (Fig 1) that combined the Fluconazole and Gly/Eth environments, chosen specifically because of their different timescales of adaptation. The first two experimental sequences were designed so that environmental blocks are periodically switched, with consecutive time spent in each on the order of the time scale of adaptation (switch_adap1 and switch_adap2): 144 consecutive generations in Gly/Eth and 48 consecutive generations in Fluconazole. The next two sequences were designed so that blocks were periodically switched at a rate that is 6-fold faster than the previous sequence; thus the consecutive time spent in each environment was 6-times shorter than the time scale of adaptation (periodic_smaller1 and periodic_smaller2): 24 consecutive generations in Gly/Eth and 8 consecutive generations in Fluconazole. We also designed one experiment with random switching between environments, with blocks for which the duration of residence is of the magnitude of the time scale of adaptation (random_adap1), and two experiments that randomly switch between blocks of environment, for which the duration of residence in each environment is less than the time scale of adaptation (random_smaller1 and random_smaller2). We also designed an experiment that combined the two block environments, i.e. SC with 2% glycerol, 2% ethanol and 4μg/ml Fluconazole (Mix–note, there is no glucose present in this environment), as well as evolved populations in either Gly/Eth, or in Fluconazole, with no switching.

**Fig 3:**
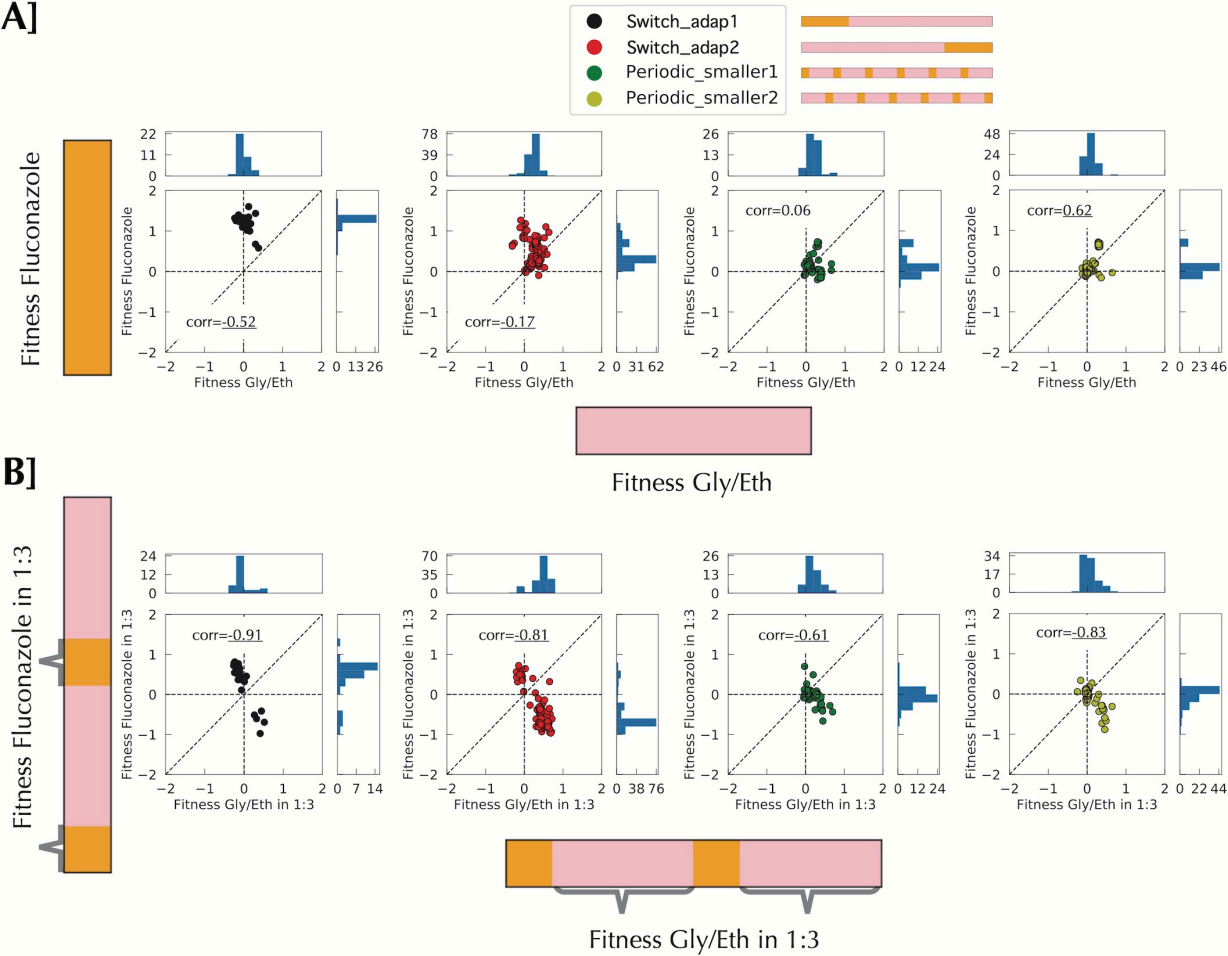
Fitness remeasurement shows different types of interaction between environments according to the time scale. All correlations are Pearson correlation. **A]** Fitness measurement for Gly/Eth and Fluconazole for 40 uninterrupted generations shows no particular correlation between environments**. B]** When fitness measurement is performed on a smaller timescale in the context of switching there is a net negative correlation between the two environments. Grey braces indicate when the fitness was measured.

Barcoded populations of diploid yeast were then evolved for 192 generations in each of the 8 different sequences of switching environments. The yeast populations contain two barcodes, such that one (BC1, low diversity) encodes the identity of the evolution experiment itself, while the second (BC2, high diversity) is used for lineage tracking within the evolution experiment, to distinguish lineages from one another. We characterized the early stages of adaptation in different dynamic environments using lineage tracking [19] to follow the population dynamics. We also isolated 336 clones from generation 192 of each evolution, determined their barcodes (see Methods), and pooled unique clones for which we could recover a barcode sequence–there were a total of 1,578 clones in this pool. To understand phenotypically how clones from different experiments had adapted to different environmental sequences, we then remeasured the fitness of all clones in this pool in 5 environments: Fluconazole, Gly/Eth, Mix, five switching cycles of 8 generations in Fluconazole and then 24 in Gly/Eth (1:3), two switching cycles of 8 generations in Fluconazole and then 8 in Gly/Eth (1:1) (Figs 3 and S1 and S2). The rationale behind remeasurement in the 1:1 environment was to determine if there has been selection for a phenotype related to their ability to *switch between* environments, instead of fitness in one of the two environment blocks *per se*. Pooled fitness remeasurement experiments were performed in triplicate as previously described [32], and also included known neutral barcoded clones, barcoded adaptive yeast from a Fluconazole only evolution and barcoded adaptive yeast from a Gly/Eth only evolution as controls. Neutral clones in our experiments are defined as clones that show behavior in the 5 environments similar to known, unevolved neutral clones (S3 Fig). Fitness was determined as described previously [33]–note, fitness is per cycle, such that in the 1:1 or the 1:3 environments, there is a single value for the cycle across both conditions, rather than two values.

### The dynamics of adaptation are affected by the environmental dynamics

Visual inspection of lineage trajectories suggested that while each sequence of environments gave rise to distinct lineage dynamics (Fig 2A), some environmental sequences gave rise to similar lineage behaviors. For example, lineage trajectories from periodic_smaller1 and periodic_smaller2 behave similarly possibly because their sequences of environment are essentially the same, except they are offset from one another by a single environmental block. Likewise, switch_adap2 and random_adap1 display visually similar lineage trajectories, likely because the lengths of the environmental blocks are similar (on the order of timescale of adaptation). By contrast, trajectories from periodic_smaller1 and periodic_smaller2 clearly differ from those of switch_adap2 and random_adap1. To further investigate similarities and differences between conditions, we classified the environments into 3 groups: strong selection, intermediate selection and weak selection, based on the change in Shannon-Wiener index (also known as Shannon’s diversity index) during the evolution (Fig 2B), and both the rate at which neutral lineages went extinct and the diversity of adaptive lineages after 192 generations (Fig 2C). Under strong selection (switch_adap1, Mix (which both initially contain Fluconazole), and random_smaller1), the diversity tends to crash early and populations are rapidly taken over by a few, fit lineages, with neutral lineages going rapidly extinct. Indeed, after 192 generations the 100 most abundant lineages are 84%, 80%, and 80% of these populations respectively. By contrast, in the weak selection environments (periodic_smaller1, periodic_smaller2) the diversity decreases around 160 generations and only a few lineages increased in frequency; after 192 generations the 100 most abundant lineages represent only 6.7% or 2.7% of the populations respectively. Under intermediate selection (switch_adap2, random_adap1 and random_smaller2) many more lineages significantly change their frequencies, while there is still diversity in the isolated lineages: the top 100 lineages at generation 192 represent 20%, 32% and 13% of the total populations for those experiments respectively. Under weak selection (periodic_smaller1 and periodic_smaller2), diversity stays high through 192 generations and only a few lineages rise in frequency. We also used the Kolmogorov-Smirnov distance between experiments (based on the fitness remeasurement data) to characterize their relatedness (Fig 2D). These groupings are largely consistent the categorization in Fig 2C, with the exception of random_smaller2, which switched between groups. In random_smaller diversity drops later than in the other conditions with which it was grouped in Fig 2C, and when it does decline, it falls precipitously, likely driven by the emergence of a highly fit lineage that is almost fixed by 192 generations (Fig 2A).

### Environmental switching can limit the increase in frequency of adaptive lineages

The fitness remeasurement data allow us to better understand the differences in evolutionary behavior between different environments, how clones evolved in one environment fare in another, and how a change in environment affects fitness and adaptation (and possibly also *evolvability*). For clones isolated from any of the environments, other than the consistent Fluconazole environment, we observe no strong deleterious fitness effects when fitness is measured in a single environment block for either of the two conditions (Figs 3A and S4). By contrast, there is a strong negative correlation between these two conditions when fitness is measured in the context of a switching environment, such that clones often display a fitness cost in the fluconazole portion of the environment (Fig 3B; for full data see S5 Fig). In Fig 3A, fitness is measured over 40 consecutive generations in each condition separately (see S1 Fig), but in Fig 3B fitness in Fluconazole is measured over 8 generations in between 24 consecutive generations in Gly/Eth, and fitness in Gly/Eth is measured following 8 generations in Fluconazole. This change of fitness behavior results in fewer beneficial lineages rising to high frequency in periodic_smaller1 and periodic_smaller2. The deleterious effect results from the 8 generations in Fluconazole rather than the 24 generations in Gly/Eth (see S6 and S7 Figs). Indeed, fitness in the 24 generations in Gly/Eth (separated by Fluconazole) and 40 generations in Gly/Eth without switching is largely the same (S7 Fig). By contrast, fitness in the Fluconazole environment over 8 generations (with a switch to Gly/Eth in between) is not strongly correlated with fitness in the Fluconazole over 40 generations with no switching (S6 Fig). The effect of environment switching is also evident in the lineage abundances, as observable ‘zig-zag’ patterns in the fitness remeasurement experiments (S1 Fig, bottom two panels). We hypothesize that at small timescales we are observing the effects of the switch rather than of the environments themselves; for example, the switch may lead to a change in lag phase, dependent on the new environmental block. Such a change then appears to slow down adaptation in these rapidly switching conditions.

### Environmental switching can result in adaptive lineages reaching high frequency

**Fig 4:**
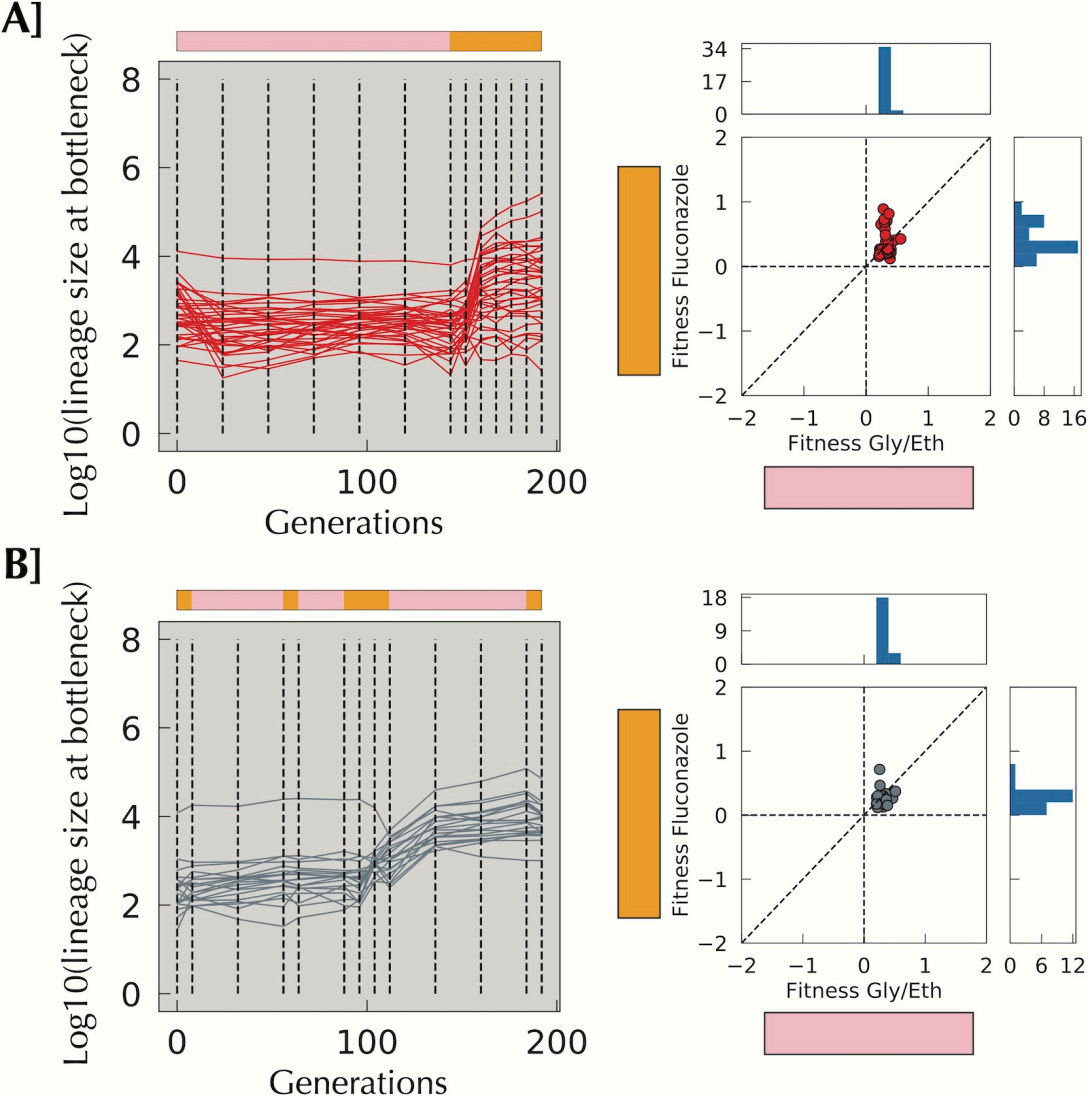
A change in the environment aids selection of lineages with high fitness in Gly/Eth. **A]** Lineages isolated from switch_adap2 having an average slope per cycle smaller than 0.08 in the Gly/Eth environment during the evolution yet have a remeasured fitness per cycle in Gly/Eth of > 0.2. Those fit Gly/Eth mutants were not able to reach high frequency in 144 generations in Gly/Eth, yet considerably increased their frequency during 48 generations in Fluconazole. **B]** A similar phenomenon is seen in Random_smaller2. The Fluconazole episode has reshuffled lineage frequencies: some frequent lineages decrease, while others increase. When the population goes back to Gly/Eth, a large increase in frequency can be seen.

**Fig 5:**
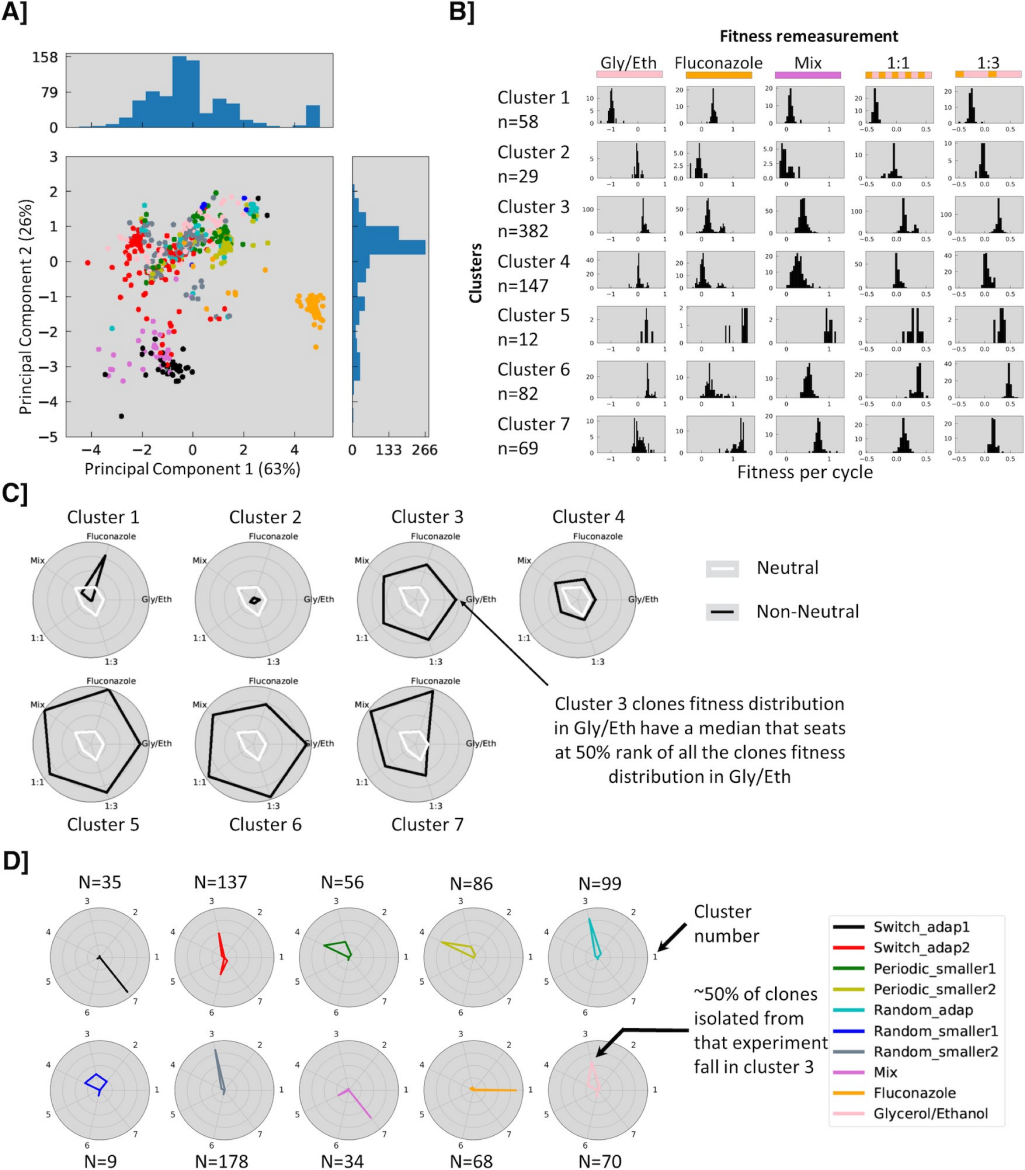
Fitness clusters. **A**] PCA analysis of combined fitness data. Each clone is represented by a five-dimensional vector of fitness values, which is projected onto a 2D space using PCA. Clones with similar fitness vectors are close together and clones from the same experiment are frequently close to one another (switch_adap2, random_smaller2, random_adap1 or fluconazole). **B]** Distribution of fitness effects in the different remeasurement experiments for each cluster (see S8C Fig for cluster membership). **C]** Spider plot of cluster characteristics in the different remeasurement experiments, indicating (in black) the percentile rank of the median fitness for each cluster in a given remeasurement environment. For example, cluster 1 is highly specialized in Fluconazole whereas cluster 5 is describing generalist behavior type. White lines indicate neutral fitness in each environment. **D]** Spider plot indicating the fraction of isolated clones in each cluster from each evolution environment.

As discussed above, sometimes environmental change can elicit a fitness cost, which can decrease the number of lineages reaching high frequency. However, in switch_adap2 and random_smaller2, a changing environment appears to result in a larger number of lineages reaching high frequency (Fig 4). In both switch_adap2 and random_smaller2, we observe little adaptation in Gly/Eth before entering the Fluconazole block, but then substantial increases in the frequencies of some lineages either at or immediately following the environment switch. These lineages show significant beneficial fitness effects in Gly/Eth (Fig 4). It is possible that selection for lineages with modest fitness benefits in the Gly/Eth condition incidentally selects generalists that also have increased fitness in Fluconazole–indeed, we see strong evidence for this, in that clones isolated from the Gly/Eth condition alone show fitness benefits in the Fluconazole fitness remeasurement consition (S4 Fig). The stronger selective pressure in the Fluconazole condition compared to Gly/Eth (see difference of scale in fitness S2B Fig) might then be the reason for this behavior, as both neutral and non-generalist lineages are then rapidly outcompeted in the face of the drug. Alternatively, some lineages may be good at switching, or instead might opportunistically take advantage of a dip in the population mean fitness due to the environmental switch. We note (see below) that in the mixed environment that generalists do indeed emerge (Fig 5), which would be consistent with the first of the above scenarios, while not ruling out the second.

### Different sequences of environment select for different phenotypes

To further understand how the sequence of environments affects the types of fitness benefits that are selected, we performed Principal Components Analysis on the fitness remeasurement data for all of the isolated mutants in the 5 remeasurement conditions; the first two principal components explain 89% of the variance (Fig 5A). Based on their fitness profiles, we defined seven clusters of clones (see Methods, S8–S10 Figs for threshold dependence), and examined the fitness of the clones in each cluster in each condition (Fig 5B and 5C). Cluster 3 contains clones that are modestly more fit in all remeasurement conditions, while Cluster 5 contains clones with extreme beneficial fitness in all the remeasurement environments, suggesting clones in both clusters are generalists. By contrast, clones in cluster 7 have very high fitness in fluconazole and the mixed environment, but generally neutral fitness in Gly/Eth. Cluster 4 clones shows fitness benefits in the mixed environment, but more modest fitness gains in the switching environments (1:1, 1:3), while clones in cluster 6 show extreme fitness gains in the switching environments (1:1, 1:3), high fitness in the Gly/Eth environment, but small/average fitness in the others. Finally, cluster 1 clones only show fitness benefits in Fluconazole, with strong trade-offs in the switching environments and the Gly/Eth environment; notably, none of the other clusters showed marked trade-offs in any of the environments. Clones from a given evolving environment map to one or occasionally two clusters (Fig 5D), while some evolving environments share some cluster usage. For example, strong initial selection for a “long” time in Fluconazole in both the Mix and switch_adap1 environments selects for similar phenotypes in cluster 7. By contrast, a “long” time in Gly/Eth followed by a “long” time in Fluconazole may explain the similar usage of cluster 3 for clones from switch_adap2, Random_adap1 and random_smaller2. Finally, cluster membership for clones from both periodic_smaller1 and periodic_smaller2 is similar (clusters 3 and 4) and shares some properties with cluster membership of clones from random_smaller1, another sequence built with blocks of 8 generations in Fluconazole.

### The dynamics of the changing environment affects both the beneficial mutational spectrum and adaptive outcomes

**Fig 6:**
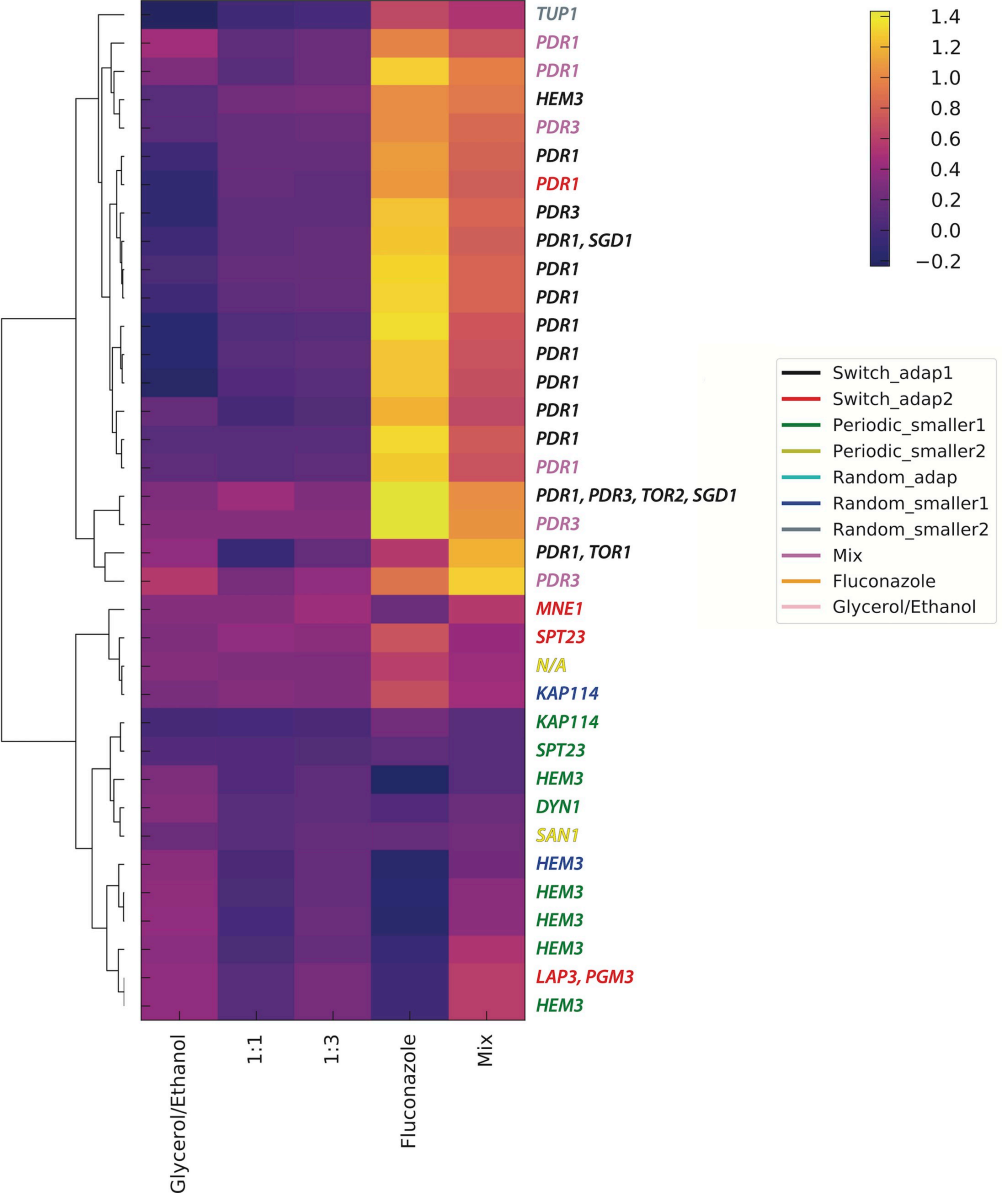
Hierarchical clustering of clone fitness data. Fitness remeasurement data, per cycle, for each clone that had reliable data in all five remeasurement conditions and at least one mutation in a gene that was recurrently mutated, were hierarchically clustered. The presumptive adaptive mutation(s) in each clone is indicated on the right, and the gene names are colored by the evolution experiment from which they were isolated.

We sequenced the whole genomes of adaptive clones isolated from generation 192 from each evolution (7 to 51 uniquely barcoded clones per environment, for a total of 198 sequenced clones; of these, 112 had reliable fitness estimates, and 81 were considered to be non-neutral in at least one of the remeasurement conditions, i.e. adaptive); across all 198 sequenced clones, we identified a total of 482 mutations. From these, we identified genes that were recurrent targets of mutation, as they are most likely to be beneficial (Table 1). The pair of paralogous zinc finger transcription factors encoded by *PDR1* and *PDR3*, mutations in which are known to result in pleiotropic drug resistance, were frequent targets of adaptation in switch_adap1, switch_adap2, and Mix, likely due to selection in a “long” consecutive period in Fluconazole. Conversely, we observed frequent, heterozygous, likely loss of function mutations in *HEM3* in the periodic_smaller1 and random_smaller1 environments, which spend “short” amounts of consecutive time in Fluconazole, and for which the main fitness contribution likely comes from Gly/Eth environment. *HEM3* encodes porphobilinogen deaminase [34], which catalyzes the third step in heme synthesis [35]. Heme is a cofactor needed for a wide variety of biological processes, including respiration and ergosterol biosynthesis; *HEM3* is essential in media lacking specific supplements and knockout mutants both lack ergosterol and fail to respire. It is unclear why decreased heme biosynthesis might be adaptive in the respiratory conditions of the Gly/Eth environment; heme is a cofactor of cytochrome C, which is responsible for the transfer of electrons between complexes III and IV in the electron transport chain. Heme is also a co-factor for cytochrome C peroxidase, which contributes to mitochondrial detoxification of hydrogen peroxide. A decreased rate of heme biosynthesis likely benefits one or both of these respiratory processes, resulting in increased fitness in the presence of a non-fermentable carbon source. Strikingly, heme is also required for sterol production (e.g. [36]), and fluconazole itself inhibits ergosterol production, through the inhibition of the heme containing protein cytochrome P450, encoded by *ERG5*. Clustering of the fitness data for the sequenced clones shows that all but one of the *HEM3* mutants show a fitness trade-off in the fluconazole containing environments, except for the Mix environment (Fig 6). This suggests that the trade-off is not due to fluconazole itself, but instead is likely due to the switch of carbon source, and that the *HEM3* loss of function fitness benefit is specific to an environment where respiration is required.

**Table 1:** Genes mutated at least twice by 2 different non-synonymous mutations (FRS = frame_shift).

	Periodic_				Random_			
Gene	adap1	adap2	smaller1	smaller2	adap	smaller1	smaller2	Mix
*PDR1*	T817K, A763E, L878S, C756S F769L, Q274R, G280V, P298L Y270S, S753C, L1056P R310W, T243A, S814Y, L537F	N234K K253E						T817K P870L N234K
*HEM3*	L318M		D218E K267(FRS) P108L G133R G250(FRS)			E65K A181D V132M		
*PDR3*	V219A H964P	L708F						K272N V954F G948S
*TMN2*	P522T			V633M				
*SGD1*	D284Y M641V							
*SPT23*		E122K	N348K					
*MNE1*		Q284L					P121S	
*LAP3*		D421N		H247Y				
*KAP114*			I107L			I335L		
*MGM1*			D841Y L626V					
*VID27*			D737A	R62K				
*SAN1*				R183K R185K(FRS)				
YHR028W-A				S73C S73A				
*DYN1*			P3506S A2554T					
*RPO31*						P507A, E516Q		

In addition to the nature of the periodic environment influencing the beneficial mutational spectrum, it also influences the nature of adaptation itself, specifically in regard to the emergence of generalists (which have positive fitness in both growth conditions) vs. specialists (which are fit in only one or a few of the growth conditions). First, we note that selection for generalists is order dependent (Fig 3). Indeed, strong selection for Fluconazole at the beginning of switch_adap1, followed by selection in Gly/Eth enriched the population for lineages with high fitness in Fluconazole but approximately neutral fitness in Gly/Eth. Conversely, growth in Gly/Eth, followed by growth in the presence of Fluconazole selects for generalists, that are highly fit in Gly/Eth, with even modest fitness benefits in Fluconazole; in addition, a few mutants with high fitness in Fluconazole, of a similar magnitude to those selected in switch_adap1, also had time to be selected (S2A and S4 Figs). This kind of generalist also arose during evolution in a consistent Gly/Eth environment (S2 and S4 Figs), despite the lack of selection in the Fluconazole environment; however, the converse is not true–clones selected in fluconazole do not show fitness gains when measured in Gly/Eth (S2 and S4 Figs), suggesting the most fit clones in Fluconazole are not generalists. Finally, a group of mutants that have high fitness in both Fluconazole and Gly/Eth arose in the Mix experiment, but those strategies were rarely observed in switch_adap1 or 2 (S2 Fig).

## Discussion

Our data demonstrate that a population’s adaptation to a changing environment depends on the order and tempo of environmental change and the strength of selection exerted by the environment. We defined our environmental sequences using two parameters: residence time in each environment and periodicity/randomness of the switches between environments. In doing so, we shed light on how those parameters influence the outcome of adaptation in dynamic environments.

Adaptation in a varying environment will also be influenced by the joint distribution of fitness effects for adaptive mutations in each of those environments–that is, the fitness effects of all beneficial mutations from any given environment as measured across the other environment(s). If there is strong antagonistic pleiotropy between two environments, then the most fit mutations in the first environment will be strongly selected against in the second environment. The evolutionary outcome will thus depend on the time scale of adaptation relative to the switching frequency–if sufficient time is spent in the first environment for adaptive mutations to reach high frequency, the second environment is likely to subsequently select for compensatory mutations that alleviate their deleterious effects. Conversely, if a short time is spent in the first environment relative to the time scale of adaptation, the second environment will instead likely cause such mutants to go extinct. In both cases, adaptation is likely to slow down. The joint distribution of fitness effects will depend on the nature of the specific environments–correlated, or even uncorrelated environments may not greatly constrain adaptation, while we expect that anticorrelated environments will.

Our study also highlights the importance of the order of environmental conditions in determining evolutionary outcomes. We explored the simplest of ordering possibilities–with only 2 environments, one ordering is simply a shift of the alternate order. Even so, we detect a strong influence (1 then 2 or 2 then 1, i.e. switch_adap1 and _2) at small time scales, probably driven by the difference of fitness scale between the two blocks in switch_adap1 and 2 (S2 Fig). The fact that we do not see any fit clones for Gly/Eth in switch_adap1 might stem from the fact that many lineages are at high frequency after the fluconazole environment: under such conditions it becomes hard to capture the rise of mutants of small to medium fitness effect, as they are swamped by the high frequency lineages. In switch_adap2, we observe the opposite: at the end of the first environment, Gly/Eth, lineages had not reached high frequencies, and they then encountered a new environment, Fluconazole, for which mutations with a much higher fitness effect could be selected. Nonetheless, we were unable to comprehensively determine *how* environment order influences adaptation. For example, environment order may become more relevant over a longer time scale, with more switches between environments, and for a population to become adapted to a well-defined, repetitive sequence of environments, the population should likely face this repeated sequence many times. Indeed, to fully understand the influence of dynamic environments on adaptation, the time scales required may be orders of magnitude longer than needed for non-switching environments. This has two main consequences for our experiment:

First, as our experimental approach relied on having barcode diversity remaining in the population, both for lineage tracking to follow the trajectories, and fitness remeasurement (we require that lineages have different barcodes to be able to remeasure their fitness in pooled fashion), by necessity we had to focus on short-term (192 generations) evolution. Indeed, we continued the evolutions for 576 generations but only one or two lineages remained. Furthermore, those lineages were already the most abundant at generation 192, meaning that lineage tracking beyond generation 192 has limited power to observe ongoing adaptation (S11 Fig). Moreover, those lineages that fix (or nearly so) by generation 576 are already “special” by generation 192, in that they are somewhat distinct from the clusters to which they belong in the PCA projection.

Second, due to the changing environment, it is challenging to measure fitness from the lineage trajectories during the evolution itself and it is therefore difficult to estimate the shape of the Distribution of Fitness Effects (DFE). Using Maximum Likelihood inference on the lineage tracking data is less informative than when analyzing such trajectories resulting from evolution in a consistent environment. This is because in our case, four models (rather than two), have to be considered, capturing the behavior of each lineage in the different conditions as either: neutral in both, neutral then adaptive, adaptive then neutral, or adaptive then further adaptive. Distinguishing between these models is challenging, as the number of data points available to reconstruct the distribution is low. Even more challenging is the uncertainty on the identity of the remeasured clone–for example, is it representative of the lineage from which it comes? We developed an algorithm for Maximum Likelihood inference of dynamics in our changing environment data that highlight the limitations of our capacity to analyze the data that way. The power and flaws of such algorithm are depicted on simulated data (S12–S20 Figs) and applied to our data (S21–S25 Figs).

Both of those limitations inherent to exploring long time scales of adaptation using barcoding approaches would likely be mitigated by using an approach that allows either periodic introduction of additional barcodes (as in [37]), or allows modification of barcodes over time (e.g. [38]), to maintain barcode diversity within the evolving populations, and measuring fitness of isolated clones in each of the environments at each environmental switch.

## Conclusions

We characterized the impacts that dynamic environments can have on adaptation and found that switching between conditions with different dynamics can influence adaptation at multiple levels. We found that the rate of adaptation itself is influenced by switching, and that adaptation could speed up or slow down, depending on the rate of switching. When switching was fast relative to the timescale of adaptation within a condition alone, adaptation was generally slowed down, while a slower switching rate *could* speed up adaption. We also found that the order of conditions influenced the adaptive outcome: that is, conditions are not commutative, similar to the idea of priority effects in the field of ecology, such that it matters what happens first. Specifically, we found that the order could influence whether generalists were selected over specialists. Finally, different targets of adaptation were selected in different dynamic environments (even when the same amount of time had been spent in each of the different conditions), necessarily resulting in different phenotypic outcomes.

## Methods

### Yeast barcode library construction

We used 8 independently constructed barcoded diploid yeast libraries (Humphrey, Hérissant et al., in prep.) each containing ~500,000 unique barcodes. Each of these libraries bears two types of barcode: a low diversity barcode that is uniquely associated to a library and a high diversity of barcodes that is associated to a specific lineage within a library. Humphrey, Hérissant et al. first introduced the low diversity barcode as part of the landing pad (see [19]), before the introduction of the high diversity barcode. Once the low diversity barcode was introduced, the high diversity barcode was incorporated for each strain carrying a different low diversity barcode separately.

### Construction of the ancestor strain

Briefly, Humphrey, Hérissant et al. (in prep.) generated the ancestral strain for barcoding by first crossing strain SHA321 [39], which carries the pre-landing pad, *Gal-Cre-NatMX*, at the *YBR209W* locus [19] and the strains HR026d (see S1 Table for genotype) which contains the Magic Marker [40].

Matα spores derived from this cross were grown on Nourseothricin, to select for the pre-landing pad, which contains *Gal-Cre-NatMX*, and then backcrossed to FY3 [41] five times, each time selecting for NatMX and Canavanine. Spores derived from the final backcross were after one more mating with FY3 was performed to obtain the diploid ancestor (Strain GSY6699). This last cross allowed us to obtain a diploid strain heterozygous for the YBR209W locus, containing one copy of the wild type locus and one copy with the pre-landing pad.

### Construction of barcoded landing pad strains

From the ancestor diploid strains, a low diversity barcoded landing pad was introduced. The landing pad contained lox66, an artificial intron, the 3’ half of *URA3* and *HygMX* along with the low diversity barcode.

To introduce this landing pad, Humphrey, Hérissant et al.(in prep.) amplified by PCR the fragment of interest from the plasmid library L001 (~75,000 barcodes) [39]. The PCR fragment was inserted into the genome by homologous recombination, using *NatMX* as a selectable marker. After selection using Hygromycin, the grown colonies, that were the Barcoded Landing pad strains, were isolated and saved for subsequent introduction of the high diversity library of DNA barcode.

### Construction of high diversity libraries and final pool

Each individual diploid barcoded landing pad was then transformed using the plasmid library pBAR3-L1 (~500,000 barcodes) [19]. This plasmid carries lox71, a DNA barcode, an artificial intron, the 5’ half of *URA3*, and *HygMX*. Transformants were plated onto SC +Gal–Ura, to allow expression of the Cre recombinase, which is under the *GAL1* promoter. The recombination between lox66 and lox71 is irreversible and brings the two barcodes in close proximity to form an intron within the complete and functional *URA3* gene. Per landing pad strain, we generated between ~10,000 and 250,000 transformants. The plates were scraped, and transformants from each plate were stored separately in glycerol at -80°C.

### Experimental evolution

The final pools, each containing ~500,000 unique barcodes, were evolved by serial batch culture in 100 mL of SC -URA media in 500 mL baffled flasks in the different sequences of environment shown in Fig 1.

In the list below, a letter represents one passage (8 generations). G stands for SC-URA 2% Ethanol/2% Glycerol (48 hours between passage), F for SC-URA 2%Glucose + 4 μg Fluconazole in 100 mL culture (24 hours between passage) and P for SC-URA 2% Ethanol/2% Glycerol 4 μg Fluconazole in 100 mL culture (48 hours between passage). All cultures were grown at 30°C; the list below corresponds to the first 192 generations.

The evolution experiments were started with a pre-culture of each pool in 100 mL of SC -URA 2% Glucose at 30°C overnight. This pre-culture was used to inoculate evolutions with ~5e7 cells (~ 400 μL).

Switch_adap1: FFFFFFGGGGGGGGGGGGGGGGGG

Switch_adap2: GGGGGGGGGGGGGGGGGGFFFFFF

Periodic_smaller1: FGGGFGGGFGGGFGGGFGGGFGGG

Periodic_smaller2: GGGFGGGFGGGFGGGFGGGFGGGF

Random_adap: GGGGGGGGGGGGGGGGGGFFFGGG

Random_smaller1: GGGFGGGGGGGGGGGGFGGGGGGF

Random_smaller2: FGGGGGGFGGGFFFGGGGGGGGGF

Mix: PPPPPPPPPPPPPPPPPPPPPPPP

Serial transfers were performed by transferring ~5e7 cells (~ 400 μL) into fresh media. The remainder of the culture was used to make glycerol stocks; 3 tubes with 1 mL of culture each were stored at -80°C (with 16.6% final Glycerol), while the remaining ~90 mL were centrifuged (3,000 rpm for 5 min) and resuspended in 5ml 0.9M sorbitol solution (0.9M Sorbitol, 0.1M Tris-HCL, pH7.5 0.1M EDTA, pH8.0) for storage at -20°C.

### PCR amplification of the barcode locus

#### DNA extraction for barcode sequencing

From the sorbitol stock, DNA was extracted using the MasterPure Yeast DNA Purification Kit (Epicentre MPY80200), with slight modifications compared to the manufacturer’s guidelines as follows: the lysis step was performed for one hour in lysis buffer, supplemented with RNAse at 1.66 μg/μL. The DNA was washed at least twice with 70% Ethanol to remove remaining contaminants. Because the number of cells in a pellet exceeded the upper limit of the kit by roughly 6-fold, 6 extractions were performed per pellet. To do so, a cell pellet was resuspended in 900 μL of the lysis buffer and aliquoted in 6 tubes (150 μL each). The aliquots were complemented with the appropriate volume of lysis buffer (150μL, 300 μL total) to follow the manufacturer’s guidelines. We used the same procedure for DNA extraction during the lineage tracking or the fitness measurements.

#### Barcode amplification from population samples

We used a two-step PCR to amplify the barcode locus for Illumina sequencing as described [19, 32], with the following modifications. For the first step, we supplemented the PCR reaction with 2mM MgCl2 and used only 6 PCR reactions per timepoint (600 ng of genomic DNA per tube). Nevertheless, in the event of PCR failure, we performed 12 additional reactions per timepoint for the first step with the same amount of DNA, lowering the DNA concentration, each with 300 ng of genomic DNA.

The primers used for this first step are listed in S2 Table. The Ns in the primers are the Unique Molecular Identifiers (UMIs) which are random nucleotides used to uniquely tag each amplicon product for subsequent removal of PCR duplicates during downstream analysis. All primers were HPLC purified to ensure that they were the correct length.

After the first step, all tubes were pooled and purified using the QIAquick PCR Purification Kit (Qiagen, 28106). For the second step, we used Herculase II Fusion DNA Polymerase (Agilent– 600677) which is a more efficient high fidelity enzyme, with the following PCR settings: 2’ 98°C, followed by 24 cycles of (10” 98°C, 20” 69°C, 30” 68°C). The PCR reaction was performed with the standard Illumina paired-end ligation primers at recommended concentrations according to the manufacturer’s guidelines. The purified first step was split into the 3 PCR reaction tubes (15 μL each).

After amplification, the tubes were pooled and the reaction was purified using one column from QIAquick PCR Purification Kit and the DNA was eluted in 30μL of water. Finally, the eluted DNA was gel-purified from a 2% agarose gel to select the appropriate band and eliminate primer dimers using the QIAquick Gel Extraction Kit. The final gel-purified DNA was quantified using Qubit fluorometry (Life Technologies).

Samples were pooled according to their concentrations.

We used the same procedure for amplification of the barcode locus during the lineage tracking or for fitness measurements.

Barcode sequencing was performed with 2x150 paired end sequencing using NextSeq.

#### Isolation of clones and fitness remeasurement

Samples from generations 192 and 576 were grown overnight in SC–URA and single cells were sorted into each well of 96 well plates containing 100 μL YPD using FACSJazz at the Stanford Shared FACS Facility as described previously [42]. We used four 96 wells plates per experiment. The barcodes for each well were recovered (see Barcode amplification of individual clones in individual wells) and a single representative for each unique barcode was pooled together. We also added 96 clones that were defined as neutral from prior fitness remeasurement experiments (Humphrey, Hérissant et al., in prep.). To have a reference to what fitness type was expected in steady environment we also added 96 clones from Fluconazole (4 μg/ml) evolution in steady environment (Humphrey, Hérissant et al., in prep.) taken at generations 48 and 96 clones from a Gly/Eth evolution (2%,2%) in consistent environment taken at generation 168 (Humphrey, Hérissant et al., in prep.).

The final pool containing all barcoded clones was grown overnight in 100 mL baffled flasks in SC–URA 2% Glucose; the ancestor was grown in a separate flask. To begin the Bulk Fitness Assay, the ancestor and the pools were each mixed in a 9:1 ratio, and then ~5e7 cells were used to inoculate cultures to remeasure fitness in each of the different environments. Each fitness remeasurement was performed in triplicate. The conditions for fitness remeasurement are as follow (S1 Fig):

- Gly/Eth: SC-URA 2% Ethanol/2% Glycerol for 40 generations with a passage at approximately every 8 generations (48 hours between passage).
- Fluconazole: SC-URA 2% Glucose + 4 μg/mL Fluconazole for 40 generations with a passage approximately every 8 generations (24 hours between passage).
- Mixture: SC-URA 2% Ethanol/2% Glycerol + 4 μg/mL Fluconazole for 40 generations with a passage approximately every 8 generations (48 hours between passage).
- 1:1: SC-URA 2% Glucose + 4 μg/mL Fluconazole for 8 generations (24 hours) then SC-URA 2% Ethanol/2% Glycerol for 8 generations (48 hours) for a total of 80 generations.
- 1:3: one passage in SC-URA 2% Glucose + 4 μg/mL Fluconazole for 8 generations (24 hours) followed by three passages in SC-URA 2% Ethanol/2% Glycerol for 8 generations (48 hours per passage) for a total of 64 generations.

#### Barcode amplification of individual clones in individual wells

To determine the locations of the individual lineages in the 96 well plates after FACS sorting, a small volume of culture was boiled and saved. For amplification, a similar 2-step protocol was used. In the first step, each well had a unique combination of primers at a final concentration of 0.416 μM. OneTaq enzyme was used for amplification following this PCR settings: 3’ 94°C—(20” 94°C, 30” 48°C, 30” 68°C) 40 cycles. After this first step, 5μL of each well were pooled into one tube per 5 plates. After centrifuging to remove cellular debris, 20μL of the pooled mix were gel purified using QIAquick Gel Extraction Kit. The purified PCR product was then diluted 50 times for the second step of the PCR. In contrast to the previously described second step, Phusion High-Fidelity DNA Polymerase was used, following manufacturer’s instructions for 12 cycles. Finally, the PCR product was gel-purified as described above and the purified product was quantified using Qubit before mixing the different libraries.

### Whole genome sequencing

#### DNA extraction

The re-arrayed plates containing clones/lineages of interest were grown in 750 mL of YPD for 2 days. DNA was extracted in 96 well format using the PureLink Pro 96 Genomic DNA Purification Kit (Thermo- K182104A). The sequencing libraries were made following the protocol previously described using Nextera technology [27,43]. We multiplexed up to 192 libraries using sets A and D primers from Nextera XT kits.

#### Analysis of whole genome sequencing data

Genome sequencing was performed with 2x150 paired end sequencing on NextSeq.

The analysis generally followed GATK best practices, as we have used previously [32,39] (code from Humphrey, Hérissant et al.). Briefly, from the split and demultiplexed fastq files, reads were trimmed for adaptors, quality and minimum length with cutadapt 1.7.1 [44]. Reads were then mapped to the yeast reference genome (*Saccharomyces_cerevisiae* R64-1-1, from SNPeff) using BWA version 0.7.10-r789 [45], variants were called with GATK’s Unified Genotyper v.3.3.0 [46] and finally the variants were annotated using SNPeff [47]. Variant filtering was performed, first by GATK recommended parameters and then using custom scripts to remove variants with low quality scores (below 150) or low coverages. Additionally, any variant in repetitive regions or low complexity regions, called using the Tandem Repeat Finder with default parameters, was excluded [48].

#### Calculation of shannon-wiener index

Shannon-Wiener index was calculated using the frequencies for all lineages as:

$$S=\sum_{k}f_{k}ln(f_{k})$$

Where, fk is the frequency of the k*th* lineage.

#### Hierarchical clustering of Kolmogorov Smirnov distances

Given two fitness distributions $F_{k}^{j}$ and $E_{l}^{j}$ for clones from experiments *k* and *l* and for which the fitness has been remeasured in remeasurement experiment *j*, we used the Kolmogorov Smirnov distance between those two distributions $D_{k,l}^{j}$, to calculate an overall distance between two experiments with $D_{k,l}=\sqrt{\sum_{j=1}^{5}{D_{k,l}^{j}}^{2}}$. Hierarchical clustering of the resulting distance matrix was performed using scipy.cluster.hierarchy module with median linkage and a max_cluster value of 4.

#### Defining clusters from fitness data

Principal Components Analysis (PCA) was performed using the sklearn.decomposition module on a five dimensional space representing the fitness data from the five remeasurement experiments. The first two principal components were used to project onto a 2-dimensional space (S3 Fig). In this projection we identified clones that occupy the same space as our neutral clones; we then defined an ellipse around the neutral clones using their spreading along the two principal components (two times the standard deviation along PC1 and PC1) and aggregated clones within this ellipse with the neutral clones and removed them from subsequent analysis. In this resulting dataset, where each point represents a non-neutral clone (S8 Fig), all the pairwise Euclidian distances between clones were calculated. This distribution of distances is multimodal, which is a sign of clustering. We chose a distance of 2.5 as our threshold to define the limits of a cluster, as it is a typical intermediate distance in our data. Finally, the matrix of Euclidian distances was hierarchically clustered using the scipy.cluster.hierarchy module. Clusters were defined as subgroup of the hierarchical matrix that were separated by more than 2.5 along the diagonal. This is identical to using a maximum cluster condition of 7 in scipy.cluster.hierarchy module. Two points were initially defining their own clusters. For the sake of simplicity, we assigned them to their closest cluster neighbors. We checked for how the choice of threshold or projection affected our clusters (S9 and S10 Figs) and determined that our clusters are generally robust to modifying these parameters.

#### Calculation of cluster characteristics

Given a fitness distribution $A_{k}^{j}$ consisting of clones from clusters k for which the fitness has been remeasured in remeasurement experiment j, and fitness distribution *A^j^* made of clones from all the clusters together for which the fitness has been remeasured in remeasurement experiment j, we calculate the characteristic of cluster k in remeasurement experiment j by calculating its median ($median(A_{k}^{j})$) and compare it to which quantile this fitness will represent in *A^j^*.

#### Fitness calculations from the fitness remeasurement experiments

The fitness of each lineage was estimated based on the slopes of the lineage trajectories, as described previously (Venkataram et al., 2016). The software repository for the fitness estimation can be found at https://github.com/barcoding-bfa/fitness-assay-python. The code was slightly modified for fitness estimation within a sequence of environments (Fluconazole 1:1, Gly/Eth 1:1, Gly/Eth 1:3), by limiting the average of the slopes and noise parameter to the slopes and noise parameter of our interest: meaning we measure either the slopes in Gly/Eth, or the slopes in Fluconazole for the two changing environment sequences that we remeasured (1:1 and 1:3).

### Simulation of adaptation in multiple environments

#### Simulations were performed as follows

500,000 barcodes are generated. They have a mean probability of 10^-5 per generation to acquire a beneficial mutation during the 16 generations of the simulated library making process. This step is performed by drawing a random number from a Poisson distribution with mean: size_of_lineage * 1e-5. The effect of that beneficial mutation is drawn from a uniform DFE corresponding to the first environment encountered.

The sizes of lineages are calculated from a Poisson statistic centered around the size of the lineage at the previous generation multiplied by their fitness advantage. This lineage is then submitted to mutation via a non-synonymous mutation rate mu_env. The same process is used as before, i.e. a random number is drawn from a Poisson distribution with mean size_of_lineage * mu_env. If multiple mutants already exist in a lineage, the next mutations occurring can only be fed by the most frequent mutant in the lineage at the beginning of an environment. In case of multiple mutations, mutation effects are considered additive.

After 8 generations, which is the estimated time of passage, the population is rescaled to its saturation size, Ns, by drawing a random number from a Poisson distribution centered around the frequency of the lineage before passage multiplied by Ns. Finally, the population goes through a bottleneck of 1/256 size reduction, again using Poisson sampling. This is repeated through the total number of passages needed.

To limit the required memory size and computation time, all mutants that reached a size of 0 (i.e. went extinct) by the time of the environment change are erased. Thus, when the environment is changed, each mutation that has not been assigned a fitness in the new environment has a probability Pn to be neutral, Pd to be deleterious and Pb to be beneficial. Practically that happens by deciding the sign of the mutation effect due to those probabilities. An absolute value for this mutation is then drawn from a uniform DFE from which the boundaries are specific to the environment. We do not assume any relationship between the two environments for a mutation’s fitness, such as correlation or anticorrelation.

Finally, for each stored time point, a sample of each lineage is draw from a Poisson distribution with mean frequency of the lineage * sequencing depth. In the presented simulations this sequencing depth per time point was 3*10^7.

## Supporting information

S1 Table (XLSX)

S2 Table (XLSX)

S1 Fig Fitness remeasurement. Each plot represents one of three replicates for lineage tracking fitness remeasurement experiments in the 5 different conditions. Dashed lines represent measured time points and the upper color strip indicates the environment. For the conditions 1:1 and 1:3, the line above the color strip indicates over which interval of environment the fitness is remeasured. Lineages known to be neutral (added in the remeasured population for that purpose) are depicted by a hollow circles. Lineages are color-coded according to their fitness proximity to either the most fit or deleterious lineage: negative fitness mutants are compared to maximally deleterious mutant and positive fitness mutants to the fittest mutants.
(TIFF)

S2 Fig Fitness remeasurement distributions in different conditions for non-neutral clones according to the experiment they are from. **A]** Rows correspond to a specific dynamic environment from which the clones were isolated. Columns correspond to the fitness distribution of those clones in the 5 remeasurement experiments. **B]** Fitness probability density distribution for the 5 different remeasurement experiments. The mean of the fitness distribution is given for each of the remeasurement experiments.
(TIFF)

S3 Fig Definition and removal of neutral clones. Each clone is represented by a 5-dimensional vector containing their fitness in the 5 fitness remeasurement environments. We then project that 5-dimensional space on a 2 dimensional space along the first two principal components. Clones known to be neutral are grouped together forming what can be approximated by an ellipse. This ellipse is defined by its spreading (twice the standard deviation) along the PC1 and PC2 axes. Clones falling inside that ellipse are considered to be neutral.
(TIFF)

S4 Fig Relationship between fitness per cycle in Fluconazole and fitness per cycle in Gly/Eth. Underlined correlation indicates a P-value<0.05.
(TIFF)

S5 Fig Relationship between fitness per cycle in Fluconazole 1:3 and fitness per cycle in Gly/Eth 1:3. Underlined correlation indicates a P-value<0.05. Braces on the color strip indicate in which block of environment fitness was remeasured.
(TIFF)

S6 Fig Relationship between fitness per cycle in Fluconazole 1:3 and fitness per cycle in Fluconazole. Underlined correlation indicates a P-value<0.05. Braces on the color strip indicate in which block of environment fitness was remeasured.
(TIFF)

S7 Fig Relationship between fitness per cycle in Gly/Eth 1:3 and fitness per cycle in Gly/Eth. Underlined correlation indicates a P-value<0.05. Braces on the color strip indicate in which block of environment fitness was remeasured.
(TIFF)

S8 Fig Cluster definition from Euclidian distance in the 2D PCA projection with threshold 2.5. **A] Hierarchical cluster matrix of Euclidian distances between clones in the PCA projection.** White dashed lines delimit clusters, defined by choosing a distance as defined below. **B] Distribution of Euclidian distances between clones in the PCA projection.** We chose a threshold of 2.5 to separate clusters. **C] Cluster coloring in the PCA projection.** Based on our clustering, clones are colored according to which cluster they fall in.
(TIFF)

S9 Fig **Effect of distance threshold on clustering A] Hierarchical cluster matrix of Euclidian distance between clones in the PCA projection, using a threshold of 1.5. B] Cluster coloring in the PCA. C] Spider plots of cluster repartitioning with different threshold.** These spider plots are very similar to **Fig 4C** and shows that the choice of the threshold is not very critical to our conclusion and fitness clustering: periodic_smaller1 and 2 still show the same usage of clusters as do periodic_adap2, random_adap1, random_smaller2, and Glycerol/Ethanol.
(TIFF)

S10 Fig Clusters from the 5-dimensional Euclidian distances. **A] Distribution of Euclidian distances between clones in the 5-dimensional space.** To calculate those distances, the fitness measurements were rescaled by their standard deviation so that they have the same weight. Here again we see different length scales and chose 1.5 as a threshold between clusters. **B] Hierarchical cluster matrix of Euclidian distance between clones in the 5-dimensional space.** With the 1.5 threshold we have 34 clusters. **C] Spider plots of clusters repartition in our evolution experiment.** The threshold choice, or even projection into a lower dimensional space does not affect our conclusions.
(TIFF)

S11 Fig Simulation of barcode diversity loss after 192 generations due to uncorrelated fitness changes between two environments. Here the two environments have the same uniform DFE [0,125], non synonymous mutation rate mu_env = 10^-5 and consecutive spent time of 96 generations. The initial barcode diversity is of 500 000 barcodes. Barcode diversity loss was calculated for simulation were Pn, Pb and Pd were varied. There is no correlated behavior of a mutant between the two environments. We see that unless high bias toward a joint distribution of fitness effect of those two environments that links preferentially a beneficial mutation in the first environment to a deleterious or neutral fitness in the second environment, we end up with a reduction of diversity of 50-fold after 192 generations.
(TIFF)

S12 Fig Simulation: Population mean fitness estimation by different mean. **A] Population mean fitness evolution in simulation: first environment.** In blue the mean fitness is calculated using a thread of similarly behaving lineages, as described in SI. In red, the mean fitness is calculated from the slopes of the lineage tracking data. In black, mean fitness is derived from exponentially decaying small size lineages, as described [19]. In yellow mean fitness is fitted using lineages for which we know the fitness. **B] Mean fitness evolution in simulation: second environment.**
(TIFF)

S13 Fig Parameter estimation from simulation with lineage mean fitness criterion and with population mean fitness calculated from thread. **All fitness are per generation. A] Sample of lineages from a simulated lineage tracking experiment**. **B] Fitness comparison between real fitness fed to the simulation and Maximum Likelihood estimation of fitness for the first environment.** The estimation includes only the top 1,000 lineages present at the end of each environment. Note, for the 2 parameter model original: a vast majority of the points are by definition stacked at coordinate (0,0), the others, only representing dozens of poorly estimated points out of 1,000. The discrepancy between estimated fitness and real fitness emphasized by the grey box, can be explained. Those boxes contain the same lineages. In the simulation for those lineages, there is no second mutation on the background of an original mutant: the two mutants coexisted before the start of the first environment because of the probability to mutate during the 16 generations of library preparation. They are not related to each other. The algorithm still predicts the correct fitness (as seen in the second upper panel of B) but was unable to choose the best model. Adding another model where the two mutants exist in the lineage before the environment where we performed the measurement does not solve the problem (S16 and S17 Figs). **C] Fitness comparison between real fitness fed to the simulation and ML estimation of fitness for the second environment.** The estimated fitness and the true fitness are well correlated but there is an offset due to the way we calculated the mean fitness. The discrepancy between real fitness and estimated fitness in the second panel comes from the fact that those mutants rose in the last ten generations of the simulation. In other words, they should not count: the algorithm should have chosen a one parameter model instead of the three parameters model. Another possibility is that some mutations actually arose but then were outcompeted by the growing population mean fitness. In that case the simulation cannot see it at the final time point, but a 3 parameter model was necessary because at some point this mutant contributed to the mean fitness of the lineage.
(TIFF)

S14 Fig Parameter estimation from simulation with Aikike criterion and with population mean fitness calculated from thread. As in S13 Fig, but with an AIC for model differentiation.
(TIFF)

S15 Fig Simulation: Full analysis from lineage mean fitness criterion and with population mean fitness calculated from thread. **A] Analysis for the first environment.** 1) Distribution of fitness effect according to the different model picked. 2) Space phase for evolution in the first environment. We can see that some of the fitness have an establishment time more negative than that allowed by their fitness. We checked if a model which would represent the trajectories as two existing mutants sharing the lineage at time zero, could alleviate this observation, but saw no improvement (S16 and S17 Figs). 3). Distribution of log likelihood for picked models. 4) Relation between the number of mutants that participate to the fitness of a lineage (non-zero at the end of the environment) and the goodness of the fit for those lineages: the worst estimations are well correlated with the hypothesis of the single mutant being broken. **B] Analysis for the second environment**. Same as **A]** but the goodness of fit 3) is now orders of magnitude worse than in panel A] and the trend with the number of mutants is even clearer. The relative poorness of the fit can be explained by our crude estimation of mean fitness, as we can see that goodness of fit comes back to acceptable level when a better estimation of mean fitness is used (S19 and S20 Figs).
(TIFF)

S16 Fig Simulation first environment: Comparison between model for fitness estimation and real fitness from the simulation with additional model: using lineage mean fitness criterion and with population mean fitness calculated from thread. **A] 2 parameters model: Original. B] 2 parameters model: Mutant.** As we removed the estimation for which the establishment time was not coherent with the fitness we see a better correlation between estimation and reality. Nonetheless, those removed points have to be estimated with the initial mix model which is a model that assume that two mutants are present in the lineage at the start of the experiment. **C] One parameter model. D] 3 parameters model initial mix of mutants: original 1 (larger fraction).** This model suffers from the same problems as the 3 parameter model with rising mutant: poor estimation of original 1.**E] 3 parameters model initial mix of mutants: original 2 (smaller fraction). F] 3 parameters model rising mutant: original.** Considering the incoherent fitness to establishment time estimation and estimate those lineages with another model, did not solve our problem: bad estimation of original mutant. G] 3 parameters rising mutant: mutant. H] Distribution of fraction for the original 2 from initial mix of mutants model. We can see that on average the initial mix mutant model predicts an initial 5 to 10% of the lineages made of the original 2, which is not negligible.
(TIFF)

S17 Fig Simulation second environment: Comparison between models for fitness estimation and real fitness from the simulation with additional models: using lineage mean fitness criterion and with population mean fitness calculated from thread. A] 2 parameters model: Original. B] 2 parameters model: Mutant. C] One parameter model. D] 3 parameters model initial mix of mutants: original 1 (larger fraction). E] 3 parameters model initial mix of mutants: original 2 (smaller fraction). F] 3 parameters model rising mutant: original. G] 3 parameters rising mutant: mutant. H] Distribution of fraction for the original 2 from 3 parameters model initial mix of mutants.
(TIFF)

S18 Fig Parameters estimation from simulation with lineage mean fitness criterion and with population mean fitness calculated from known lineage fitness. (TIFF)

S19 Fig **Overall comparison of the fits in environment 1 of simulation, of the first 1,000 largest lineages at the end of environment 1: A] Using for mean population fitness estimation either the thread method or B] Known lineages’ fitness.** Using the population mean fitness calculated from known lineage fitness strongly increases the goodness of fit (comparing panels A]3) and B]3)). In terms of distribution of the different parameters there is no striking differences even though the two ways of calculating mean population fitness show sensible differences.
(TIFF)

S20 Fig **Overall comparison of the fits in environment 2 of simulation, of the first 1,000 biggest lineages at the end of environment 2: A] Using the thread method or B] Known lineages fitness for estimation of the population mean fitness.** Using the mean fitness from known lineage fitness strongly increase the goodness of fit (comparing panels A]3) and B]3)). In terms of distribution of the different parameters there is no striking differences even though the two ways of calculating mean population fitness shows sensible difference (S12 Fig).
(TIFF)

S21 Fig Recapitulation of lineage tracking analysis for switch_adap1 evolution using a model choice based on lineage fitness criterion and a population mean fitness calculated from thread. **A] Analysis of the top 1,000 largest lineages at the end of the first environment.** The most left panel is an estimation of the evolution of the population mean fitness using the exponential decay of lineages behaving similarly (thread). The population mean fitness function has a big jump in the middle, which follows well the type of behavior that we see in the lineage tracking, but which is not a behavior expected for usual population mean fitness. 1) Distribution of fitness effects according to the different model picked. The underlined number on the right of each panel represents the fraction of this particular model chosen by the algorithm. 2) Space phase for evolution in the first environment. 3) Fitness comparison between measured fitness and Maximum Likelihood estimation of fitness for the first environment of switch_adap1. The estimation follows quite well the measured fitness with of course an offset that comes from our estimation of mean fitness. 4) Distribution of log-likelihood for picked models. The fits are not very good as they are peaked around -10^4, whereas the good fit usually peaked around -10^2 (see simulations). This is probably coming from the big jump in mean fitness that we cannot explain. 5) Relationship between the size of a lineage at the beginning of an environment (as a proxy for the number of mutants contributing to the mean fitness of the lineage) and the goodness of the fit for those lineages. L is the likelihood of the model. **B] Analysis of the top 1,000 largest lineages at the end of the second environment.** Everything is smoother and makes more sense for the second environment. Still the goodness of fit is quite bad and should be reestimated using population mean fitness estimated from known lineages.
(TIFF)

S22 Fig Recapitulation of lineage tracking analysis for periodic adap2 evolution using a model choice based on lineage fitness and a population mean fitness calculated from thread. (TIFF)

S23 Fig Recapitulation of lineage tracking analysis for second environment of periodic adap1 and 2 evolution using a model choice based on lineage mean fitness and a population mean fitness calculated from known lineages. **A]** The goodness of fit distribution 3) is orders of magnitude better than with the thread way to calculate population mean fitness. In addition, most of the 1 parameter models seen before have been moved to a three parameters model. **B]** The goodness of fit distribution 3) is orders of magnitude better than before even though still being quite large. If one looks at the mean population function associated with that Fluconazole environment in all our experiments, it is obvious that we are lacking a full description of that environment.
(TIFF)

S24 Fig Recapitulation of lineage tracking analysis for periodic_smaller2 evolution using a model choice based on lineage fitness and a population mean fitness calculated from thread. (TIFF)

S25 Fig Aikike criterion and thread mean fitness: Comparison of fitness between remeasurement and Maximum Likelihood estimation from lineage tracking. (TIFF)
