# Supplementary figures and images for: Adaptation is influenced by the complexity of environmental change during evolution in a dynamic environment
Source: PLoS Genet. 2021 Jan 25;17(1):e1009314. doi: 10.1371/journal.pgen.1009314 (PMC7861553; doi:10.1371/journal.pgen.1009314)

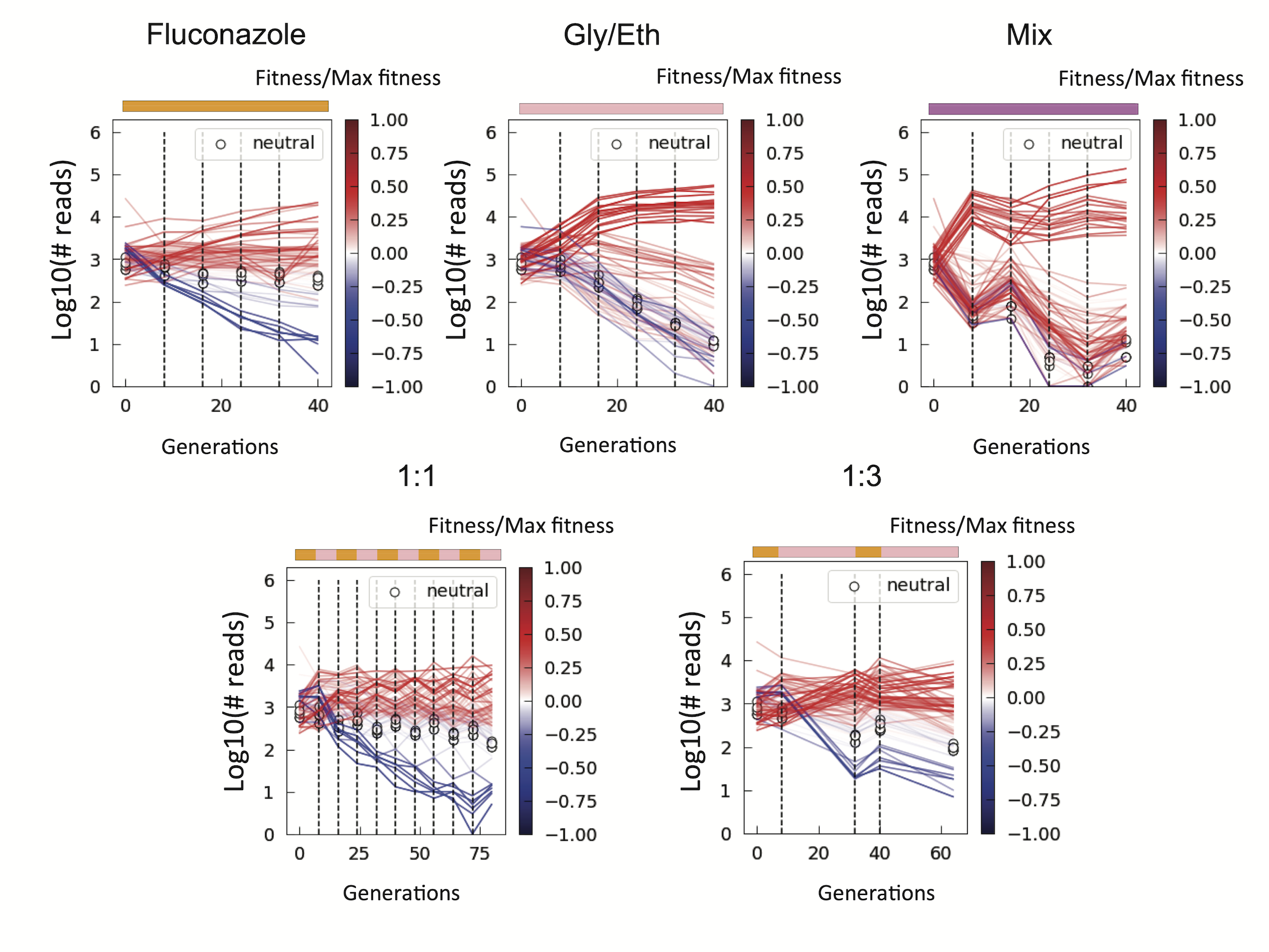

Supplement: S1 Fig — Each plot represents one of three replicates for lineage tracking fitness remeasurement experiments in the 5 different conditions. Dashed lines represent measured time points and the upper color strip indicates the environment. For the conditions 1:1 and 1:3, the line above the color strip indicates over which interval of environment the fitness is remeasured. Lineages known to be neutral (added in the remeasured population for that purpose) are depicted by a hollow circles. Lineages are color-coded according to their fitness proximity to either the most fit or deleterious lineage: negative fitness mutants are compared to maximally deleterious mutant and positive fitness mutants to the fittest mutants. (TIFF) [file pgen.1009314.s003.tiff]

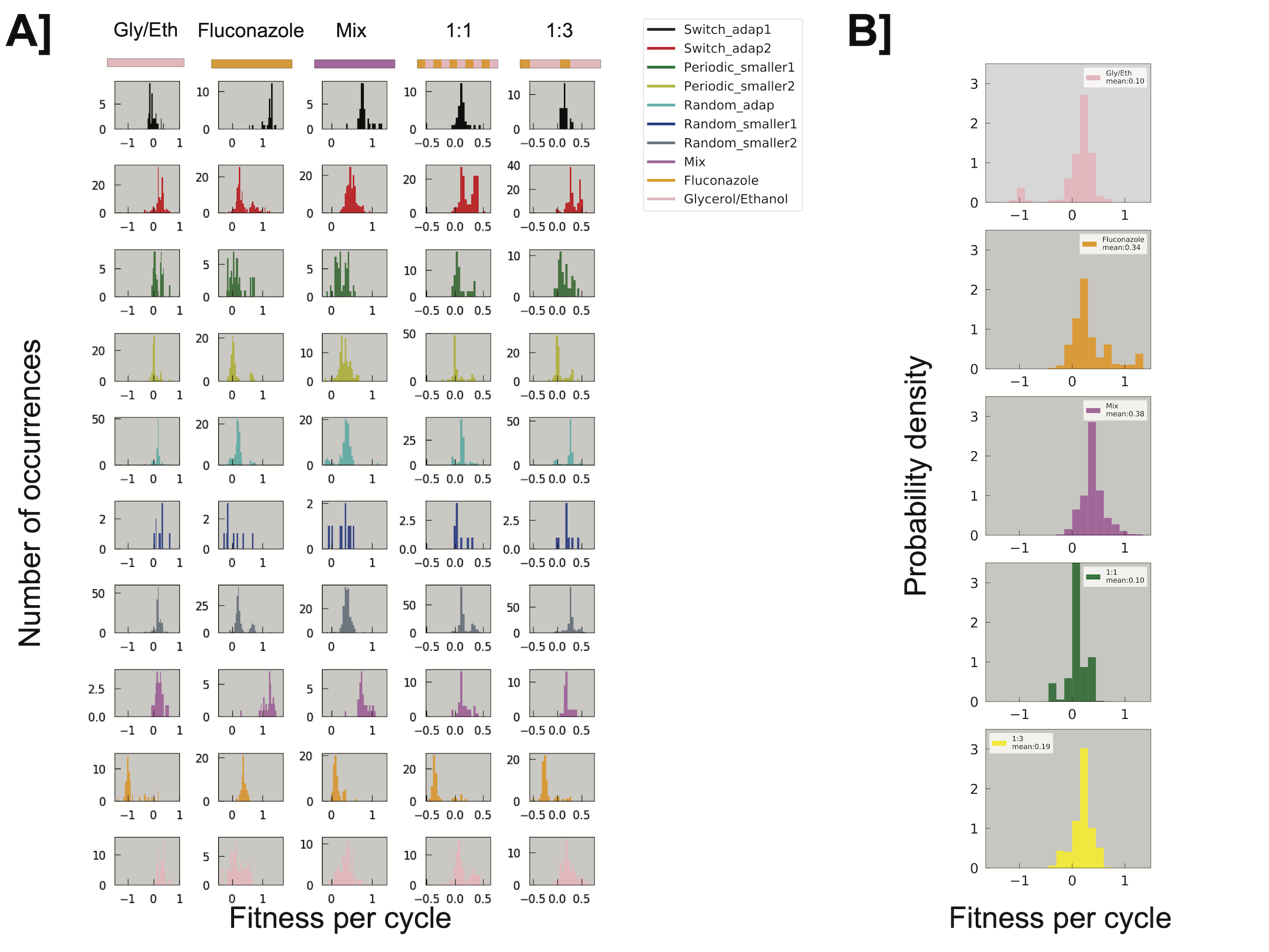

Supplement: S2 Fig — A] Rows correspond to a specific dynamic environment from which the clones were isolated. Columns correspond to the fitness distribution of those clones in the 5 remeasurement experiments. B] Fitness probability density distribution for the 5 different remeasurement experiments. The mean of the fitness distribution is given for each of the remeasurement experiments. (TIFF) [file pgen.1009314.s004.tiff]

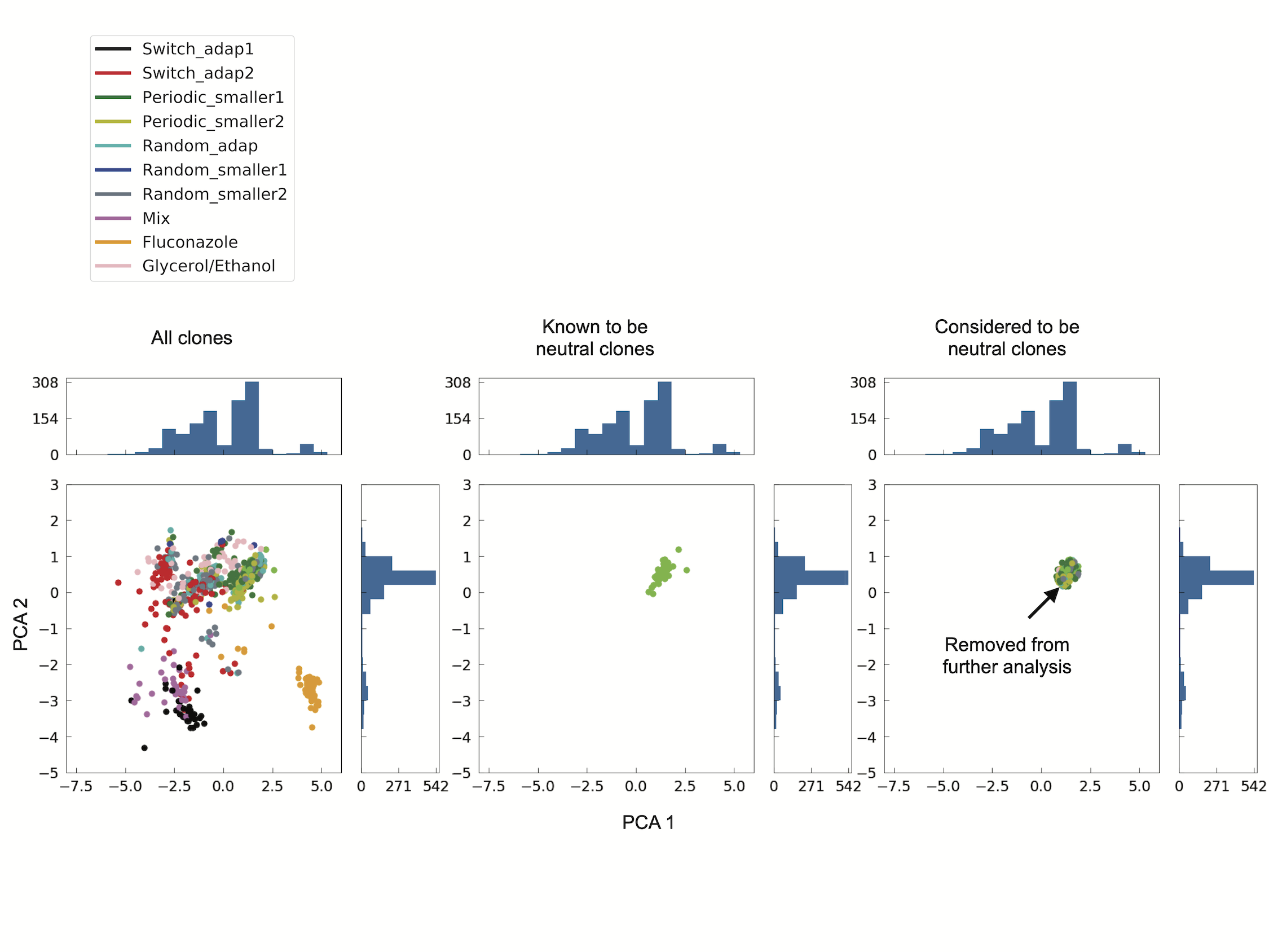

Supplement: S3 Fig — Each clone is represented by a 5-dimensional vector containing their fitness in the 5 fitness remeasurement environments. We then project that 5-dimensional space on a 2 dimensional space along the first two principal components. Clones known to be neutral are grouped together forming what can be approximated by an ellipse. This ellipse is defined by its spreading (twice the standard deviation) along the PC1 and PC2 axes. Clones falling inside that ellipse are considered to be neutral. (TIFF) [file pgen.1009314.s005.tiff]

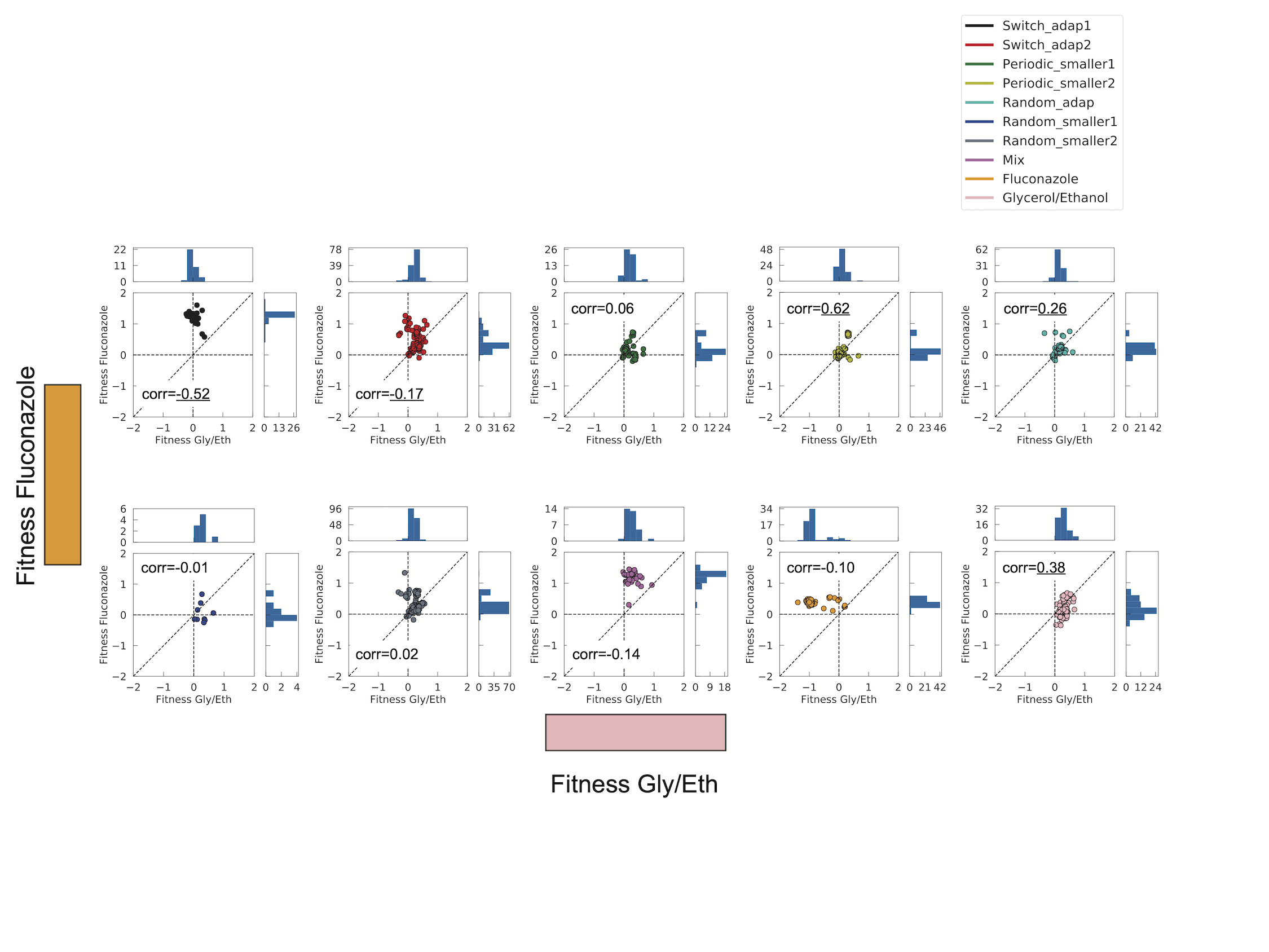

Supplement: S4 Fig — Underlined correlation indicates a P-value<0.05. (TIFF) [file pgen.1009314.s006.tiff]

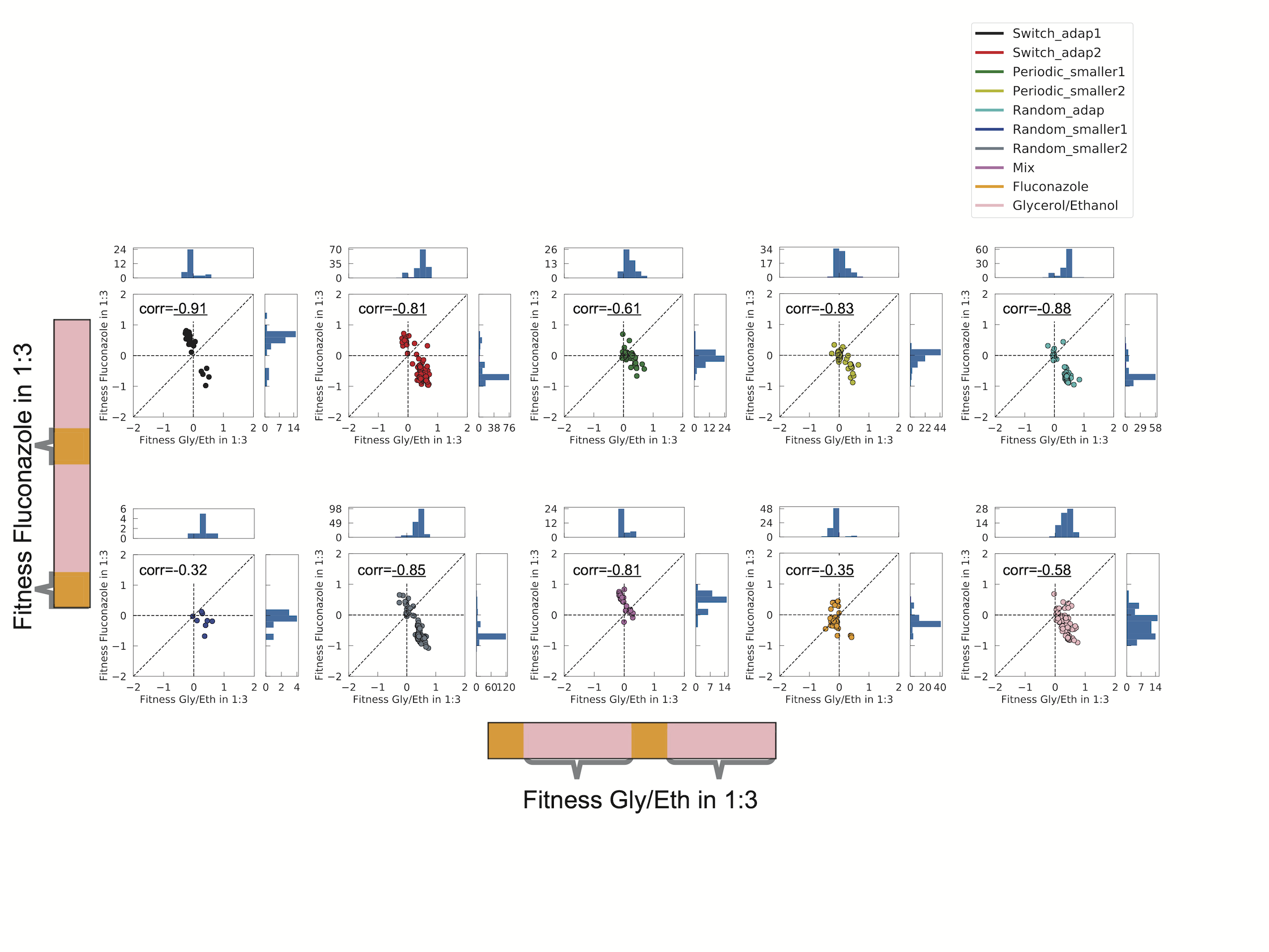

Supplement: S5 Fig — Underlined correlation indicates a P-value<0.05. Braces on the color strip indicate in which block of environment fitness was remeasured. (TIFF) [file pgen.1009314.s007.tiff]

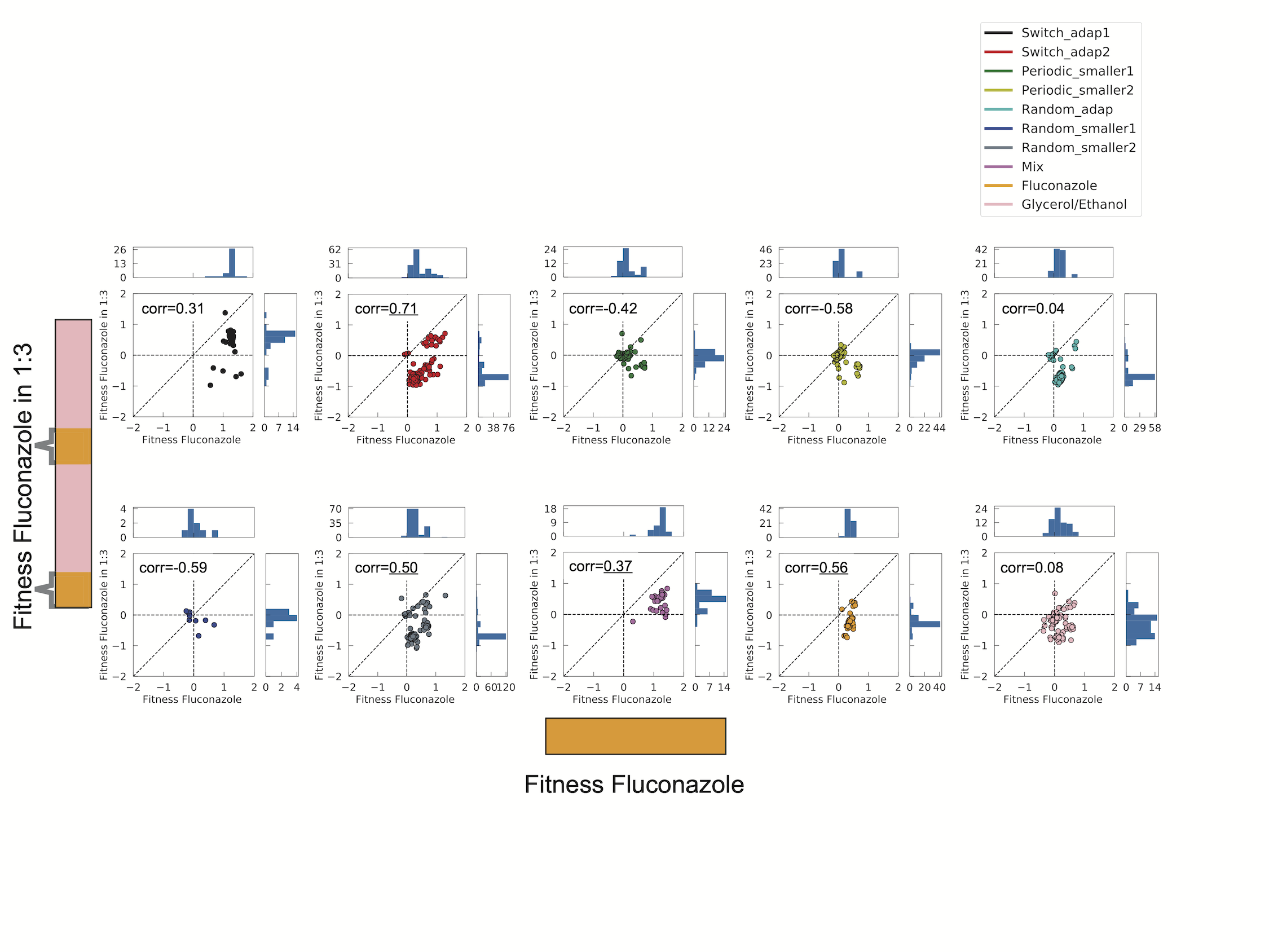

Supplement: S6 Fig — Underlined correlation indicates a P-value<0.05. Braces on the color strip indicate in which block of environment fitness was remeasured. (TIFF) [file pgen.1009314.s008.tiff]

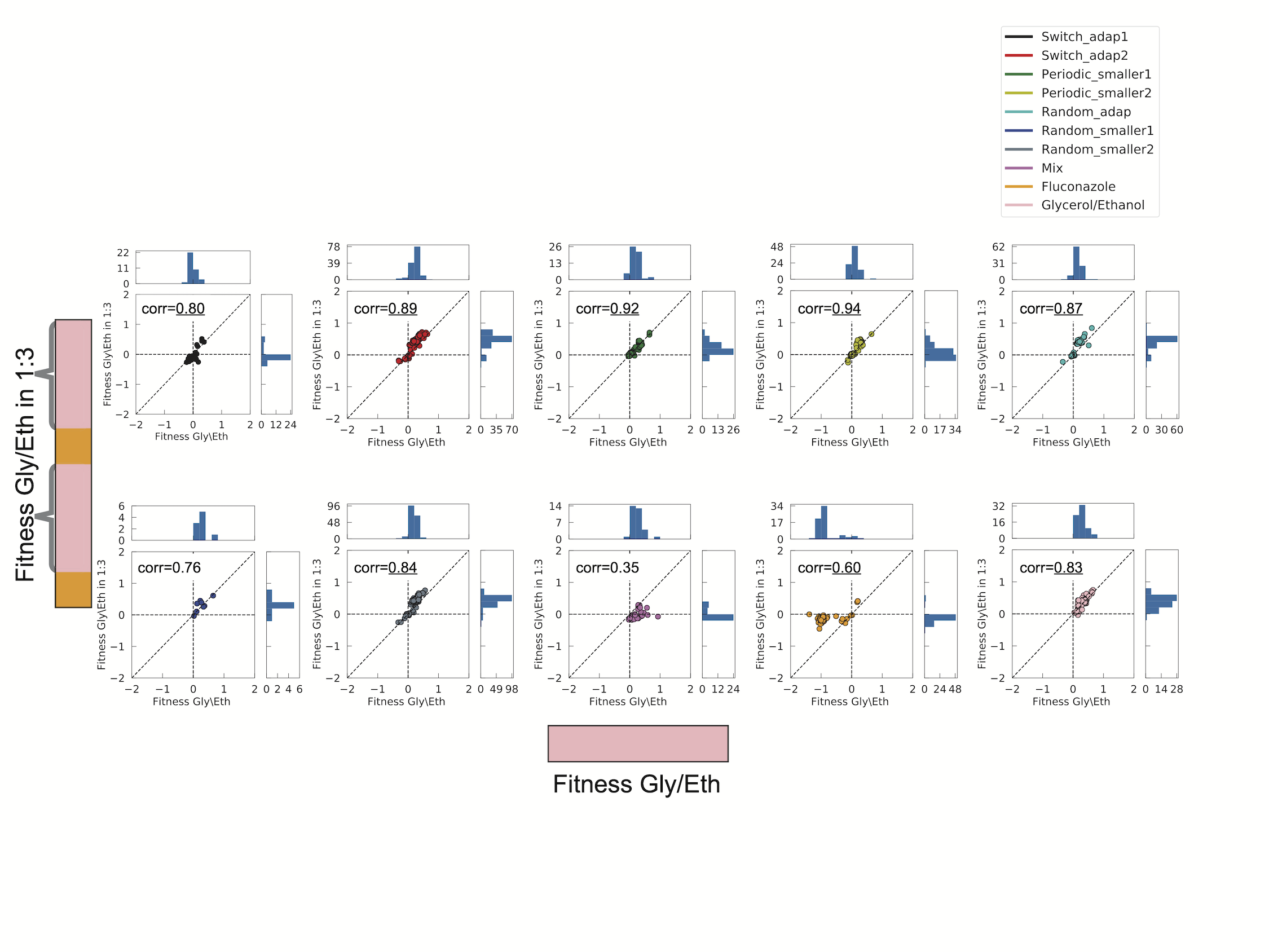

Supplement: S7 Fig — Underlined correlation indicates a P-value<0.05. Braces on the color strip indicate in which block of environment fitness was remeasured. (TIFF) [file pgen.1009314.s009.tiff]

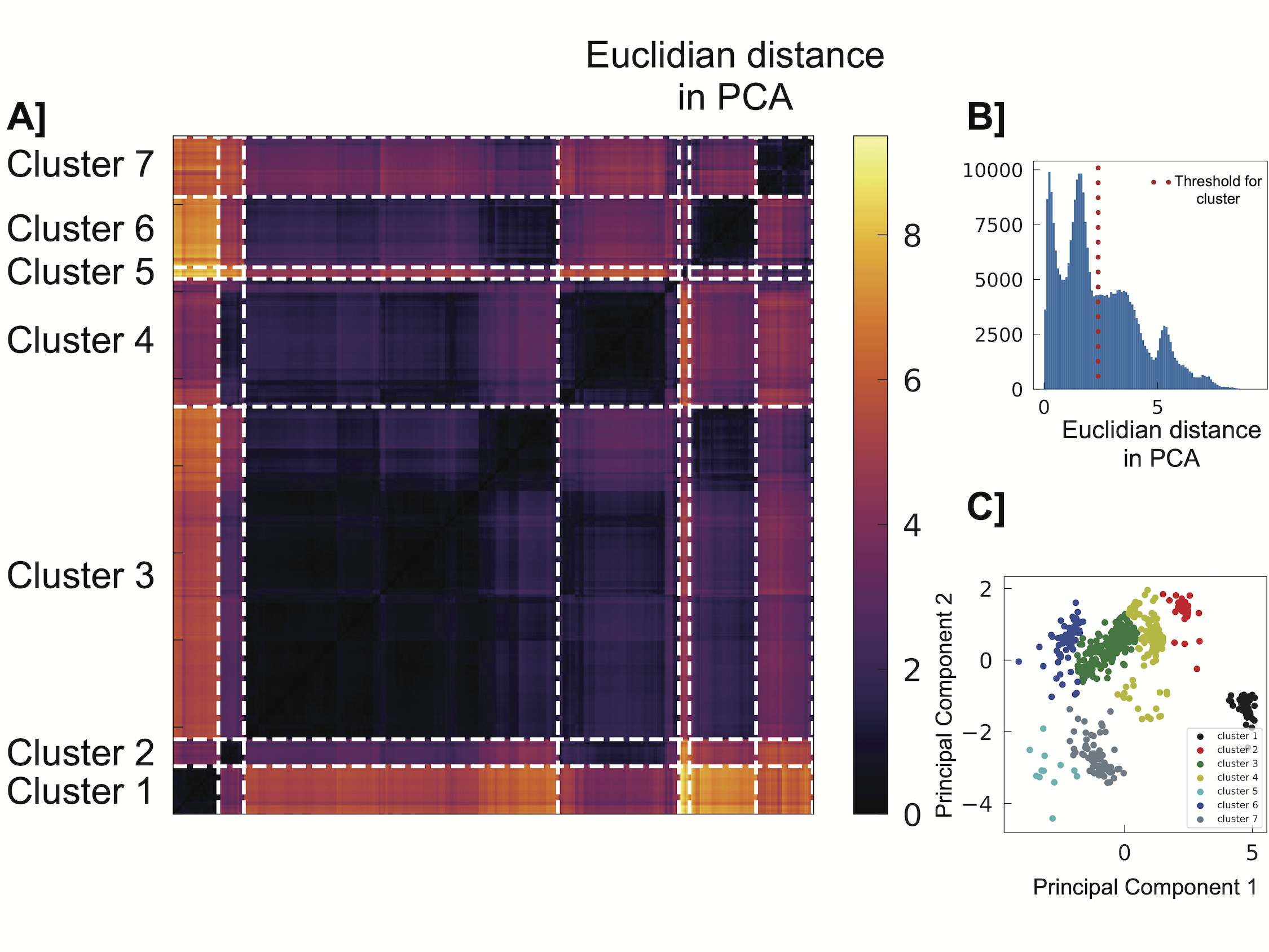

Supplement: S8 Fig — A] Hierarchical cluster matrix of Euclidian distances between clones in the PCA projection. White dashed lines delimit clusters, defined by choosing a distance as defined below. B] Distribution of Euclidian distances between clones in the PCA projection. We chose a threshold of 2.5 to separate clusters. C] Cluster coloring in the PCA projection. Based on our clustering, clones are colored according to which cluster they fall in. (TIFF) [file pgen.1009314.s010.tiff]

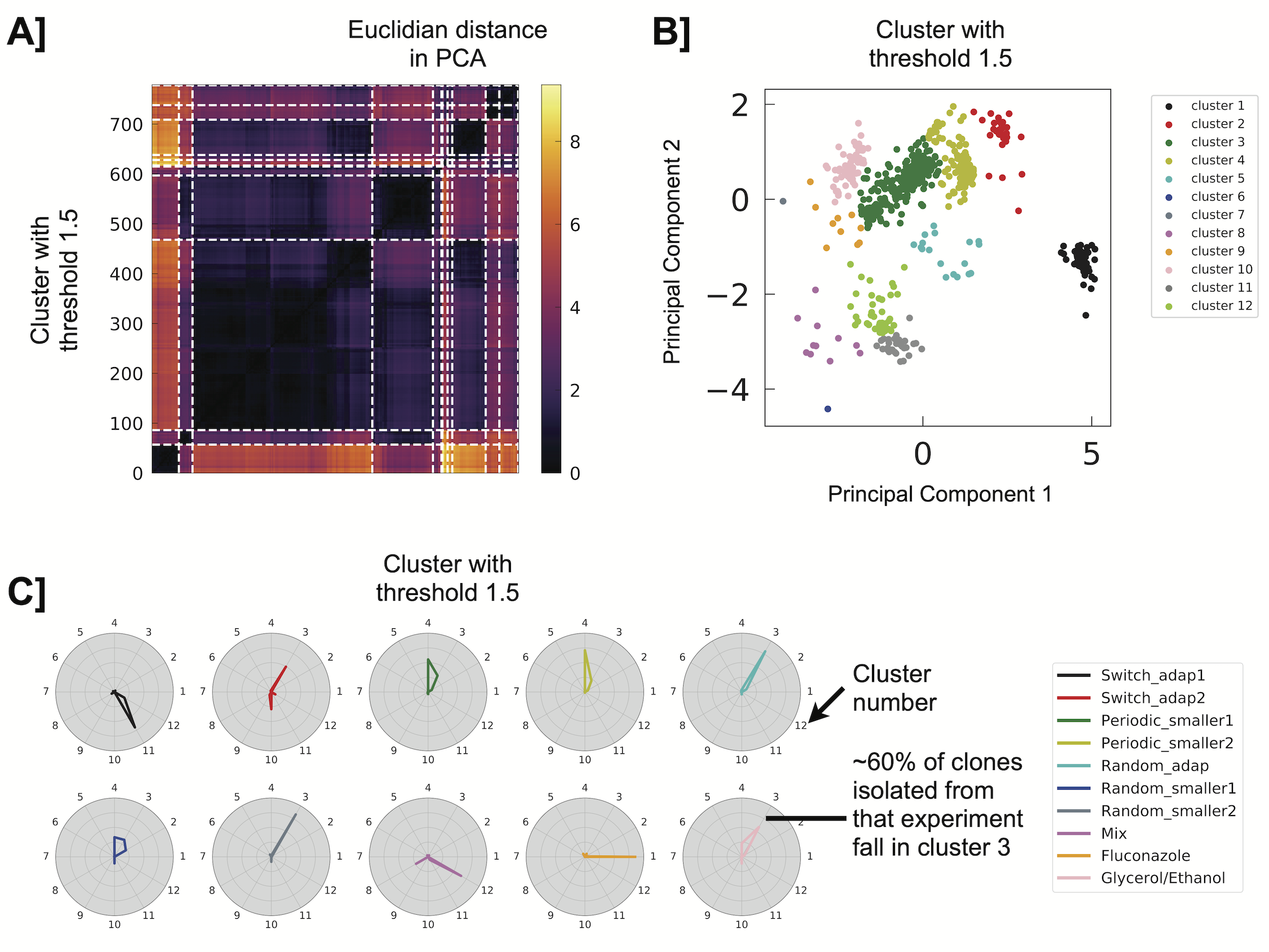

Supplement: S9 Fig — Effect of distance threshold on clustering A] Hierarchical cluster matrix of Euclidian distance between clones in the PCA projection, using a threshold of 1.5. B] Cluster coloring in the PCA. C] Spider plots of cluster repartitioning with different threshold. These spider plots are very similar to Fig 4C and shows that the choice of the threshold is not very critical to our conclusion and fitness clustering: periodic_smaller1 and 2 still show the same usage of clusters as do periodic_adap2, random_adap1, random_smaller2, and Glycerol/Ethanol. (TIFF) [file pgen.1009314.s011.tiff]

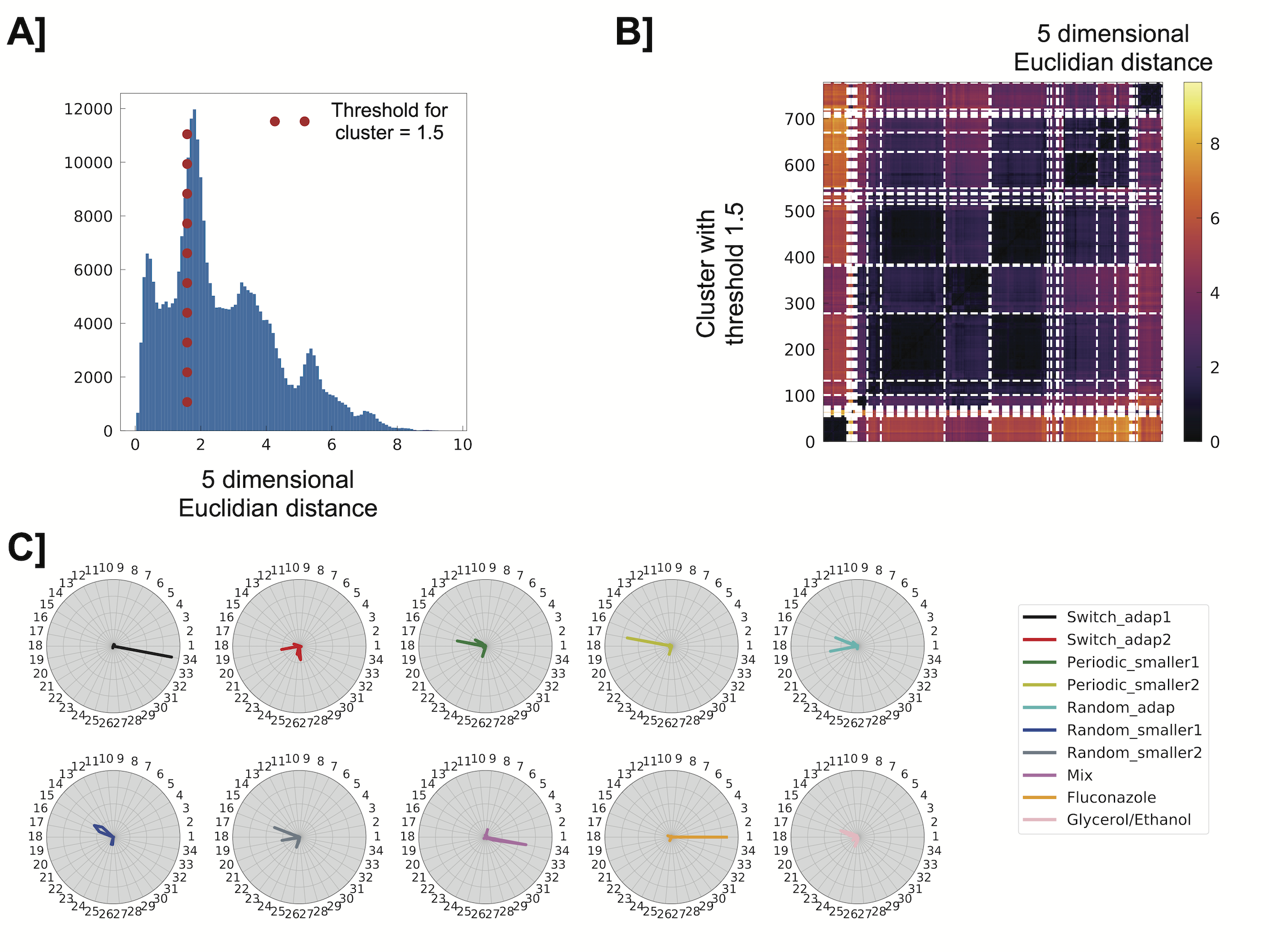

Supplement: S10 Fig — A] Distribution of Euclidian distances between clones in the 5-dimensional space. To calculate those distances, the fitness measurements were rescaled by their standard deviation so that they have the same weight. Here again we see different length scales and chose 1.5 as a threshold between clusters. B] Hierarchical cluster matrix of Euclidian distance between clones in the 5-dimensional space. With the 1.5 threshold we have 34 clusters. C] Spider plots of clusters repartition in our evolution experiment. The threshold choice, or even projection into a lower dimensional space does not affect our conclusions. (TIFF) [file pgen.1009314.s012.tiff]

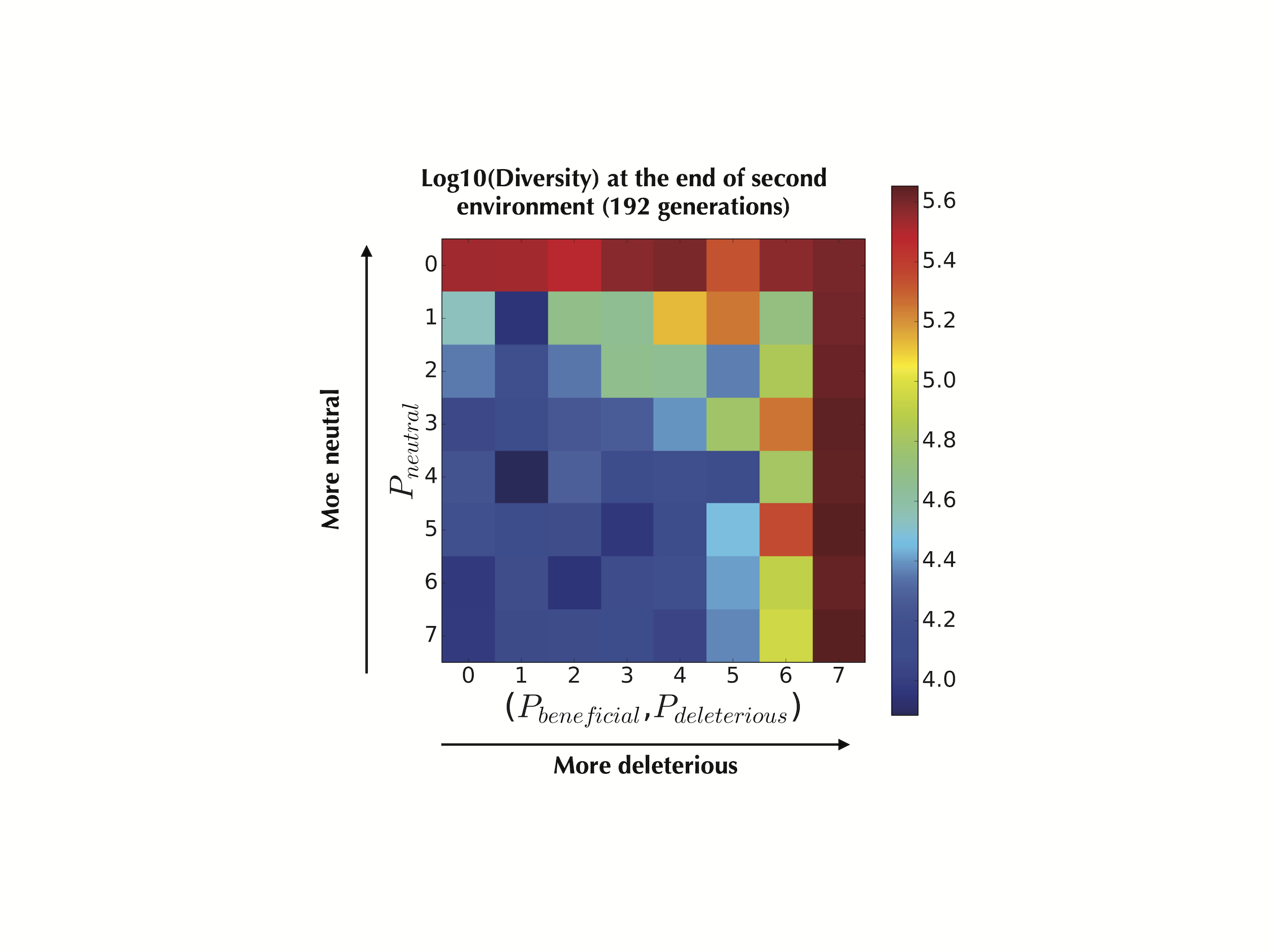

Supplement: S11 Fig — Here the two environments have the same uniform DFE [0,125], non synonymous mutation rate mu_env = 10^-5 and consecutive spent time of 96 generations. The initial barcode diversity is of 500 000 barcodes. Barcode diversity loss was calculated for simulation were Pn, Pb and Pd were varied. There is no correlated behavior of a mutant between the two environments. We see that unless high bias toward a joint distribution of fitness effect of those two environments that links preferentially a beneficial mutation in the first environment to a deleterious or neutral fitness in the second environment, we end up with a reduction of diversity of 50-fold after 192 generations. (TIFF) [file pgen.1009314.s013.tiff]

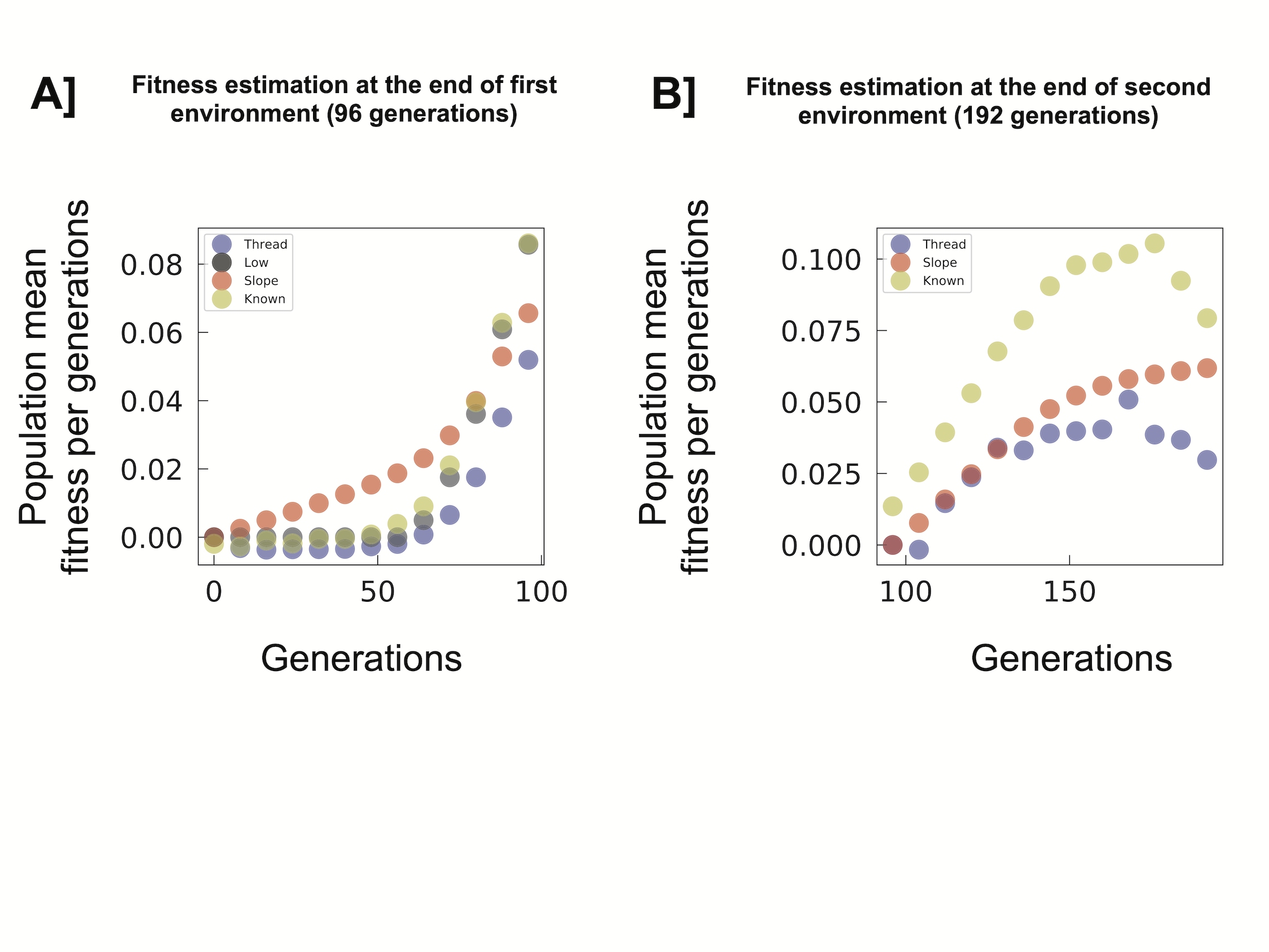

Supplement: S12 Fig — A] Population mean fitness evolution in simulation: first environment. In blue the mean fitness is calculated using a thread of similarly behaving lineages, as described in SI. In red, the mean fitness is calculated from the slopes of the lineage tracking data. In black, mean fitness is derived from exponentially decaying small size lineages, as described [19]. In yellow mean fitness is fitted using lineages for which we know the fitness. B] Mean fitness evolution in simulation: second environment. (TIFF) [file pgen.1009314.s014.tiff]

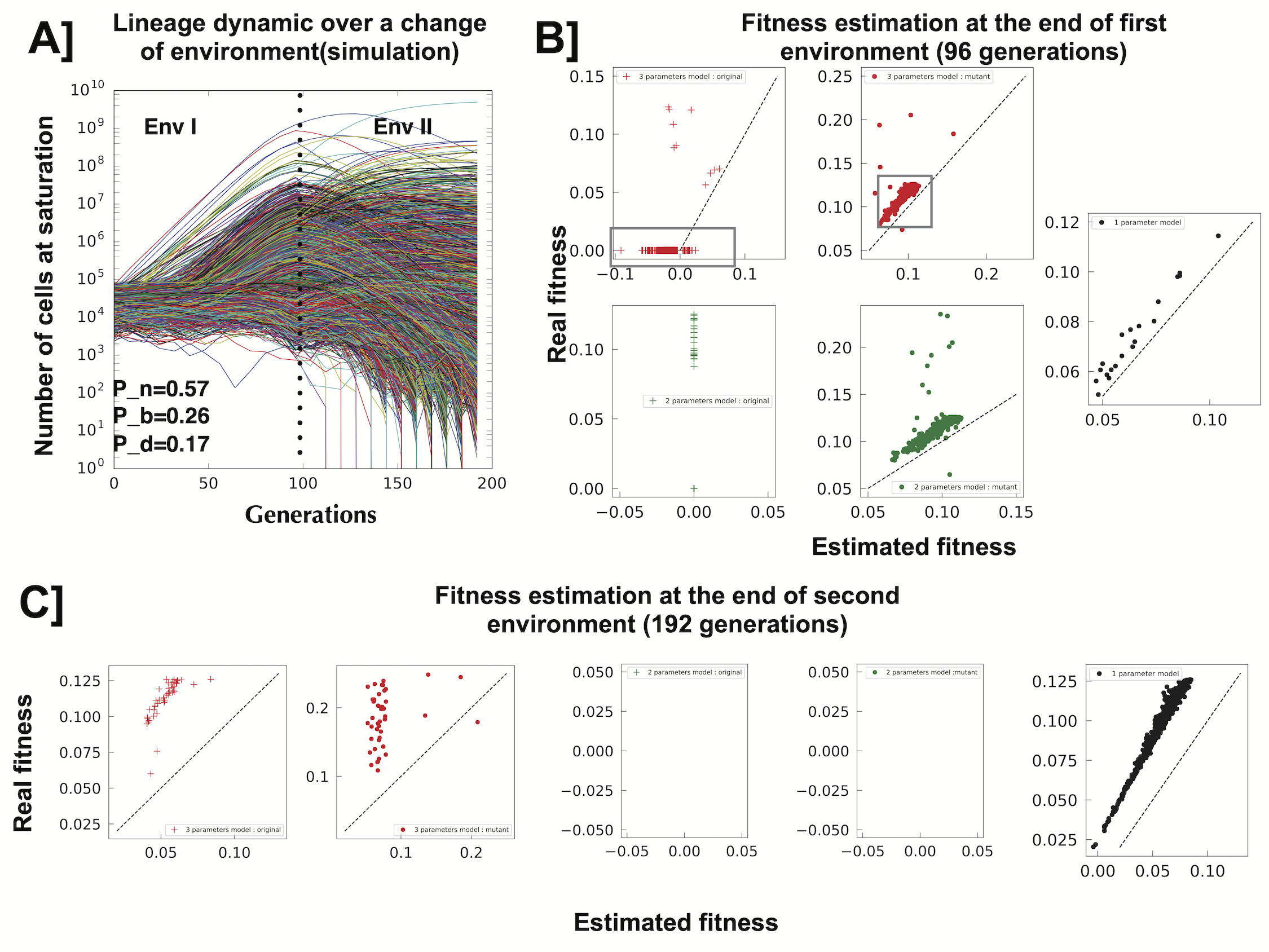

Supplement: S13 Fig — All fitness are per generation. A] Sample of lineages from a simulated lineage tracking experiment. B] Fitness comparison between real fitness fed to the simulation and Maximum Likelihood estimation of fitness for the first environment. The estimation includes only the top 1,000 lineages present at the end of each environment. Note, for the 2 parameter model original: a vast majority of the points are by definition stacked at coordinate (0,0), the others, only representing dozens of poorly estimated points out of 1,000. The discrepancy between estimated fitness and real fitness emphasized by the grey box, can be explained. Those boxes contain the same lineages. In the simulation for those lineages, there is no second mutation on the background of an original mutant: the two mutants coexisted before the start of the first environment because of the probability to mutate during the 16 generations of library preparation. They are not related to each other. The algorithm still predicts the correct fitness (as seen in the second upper panel of B) but was unable to choose the best model. Adding another model where the two mutants exist in the lineage before the environment where we performed the measurement does not solve the problem (S16 and S17 Figs). C] Fitness comparison between real fitness fed to the simulation and ML estimation of fitness for the second environment. The estimated fitness and the true fitness are well correlated but there is an offset due to the way we calculated the mean fitness. The discrepancy between real fitness and estimated fitness in the second panel comes from the fact that those mutants rose in the last ten generations of the simulation. In other words, they should not count: the algorithm should have chosen a one parameter model instead of the three parameters model. Another possibility is that some mutations actually arose but then were outcompeted by the growing population mean fitness. In that case the simulation cannot see it at the f [file pgen.1009314.s015.tiff]

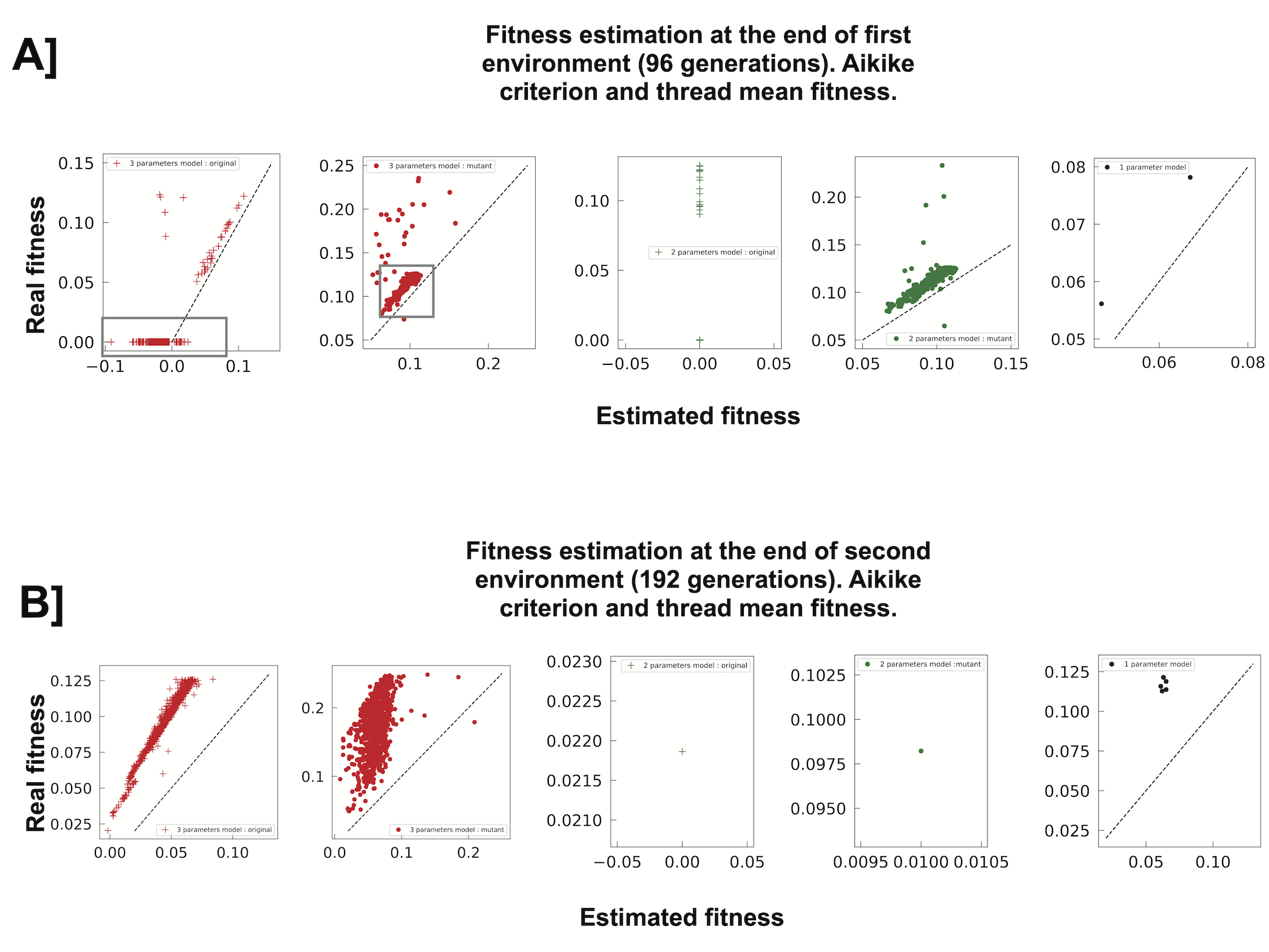

Supplement: S14 Fig — As in S13 Fig, but with an AIC for model differentiation. (TIFF) [file pgen.1009314.s016.tiff]

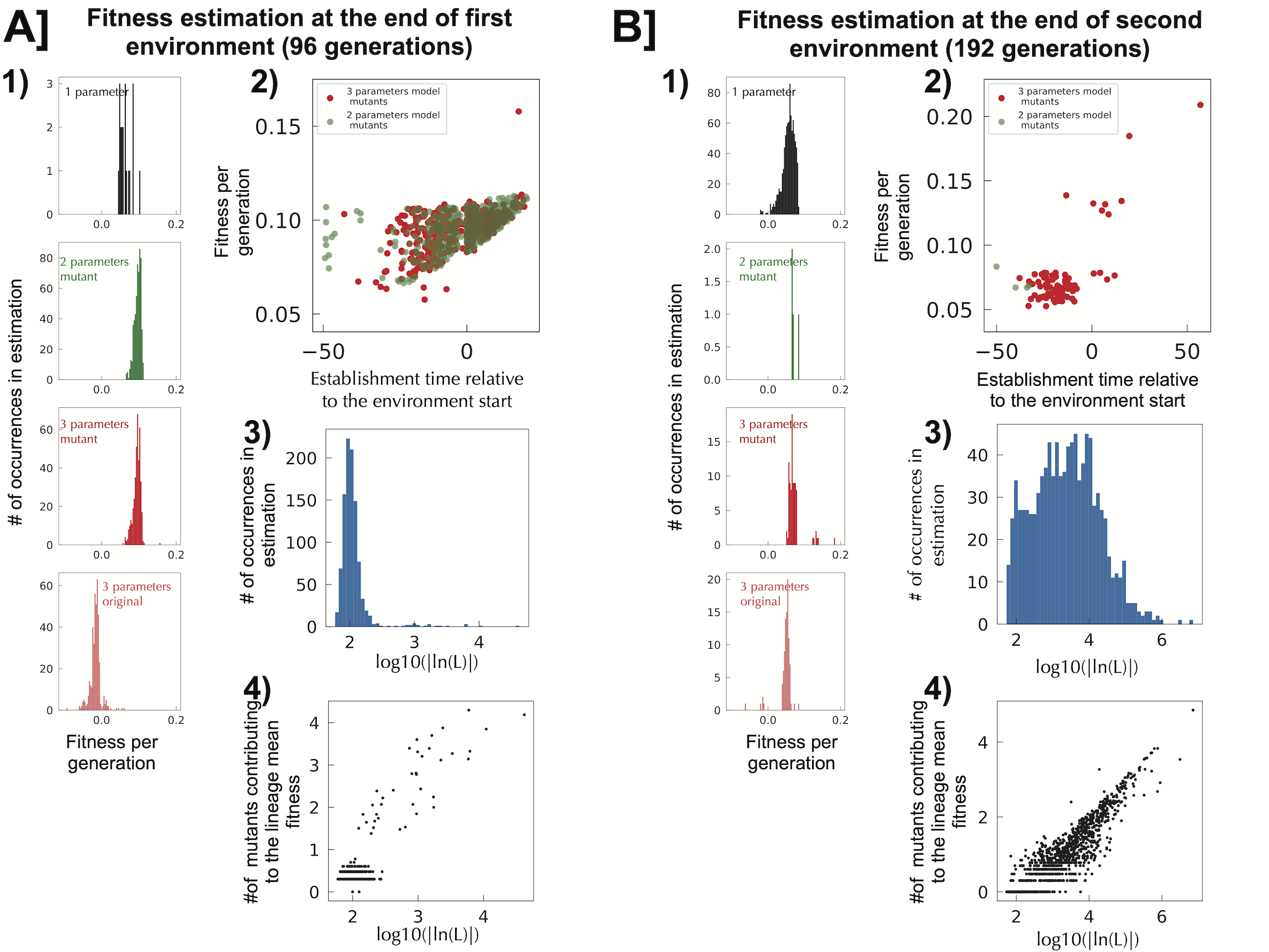

Supplement: S15 Fig — A] Analysis for the first environment. 1) Distribution of fitness effect according to the different model picked. 2) Space phase for evolution in the first environment. We can see that some of the fitness have an establishment time more negative than that allowed by their fitness. We checked if a model which would represent the trajectories as two existing mutants sharing the lineage at time zero, could alleviate this observation, but saw no improvement (S16 and S17 Figs). 3). Distribution of log likelihood for picked models. 4) Relation between the number of mutants that participate to the fitness of a lineage (non-zero at the end of the environment) and the goodness of the fit for those lineages: the worst estimations are well correlated with the hypothesis of the single mutant being broken. B] Analysis for the second environment. Same as A] but the goodness of fit 3) is now orders of magnitude worse than in panel A] and the trend with the number of mutants is even clearer. The relative poorness of the fit can be explained by our crude estimation of mean fitness, as we can see that goodness of fit comes back to acceptable level when a better estimation of mean fitness is used (S19 and S20 Figs). (TIFF) [file pgen.1009314.s017.tiff]

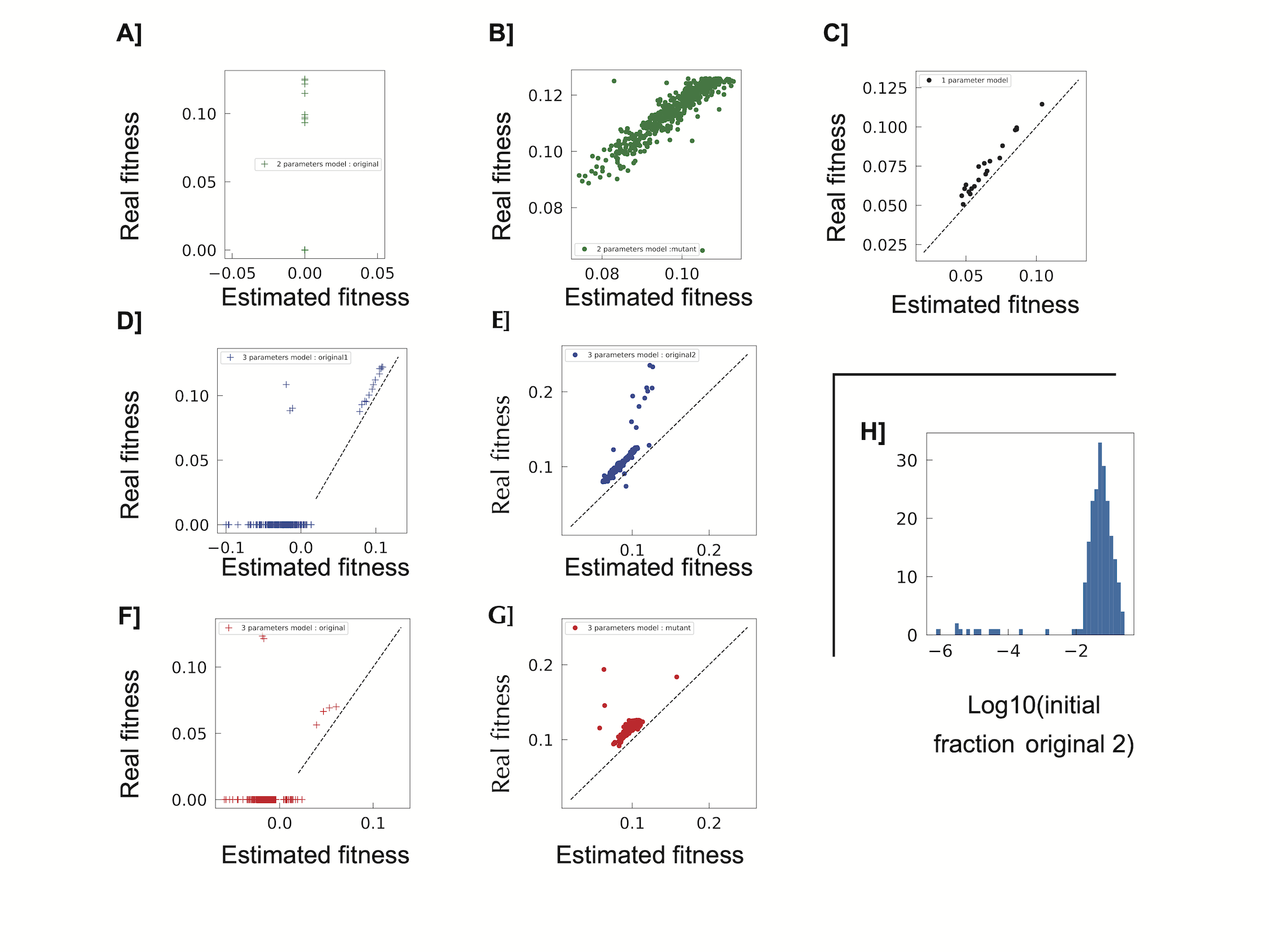

Supplement: S16 Fig — A] 2 parameters model: Original. B] 2 parameters model: Mutant. As we removed the estimation for which the establishment time was not coherent with the fitness we see a better correlation between estimation and reality. Nonetheless, those removed points have to be estimated with the initial mix model which is a model that assume that two mutants are present in the lineage at the start of the experiment. C] One parameter model. D] 3 parameters model initial mix of mutants: original 1 (larger fraction). This model suffers from the same problems as the 3 parameter model with rising mutant: poor estimation of original 1.E] 3 parameters model initial mix of mutants: original 2 (smaller fraction). F] 3 parameters model rising mutant: original. Considering the incoherent fitness to establishment time estimation and estimate those lineages with another model, did not solve our problem: bad estimation of original mutant. G] 3 parameters rising mutant: mutant. H] Distribution of fraction for the original 2 from initial mix of mutants model. We can see that on average the initial mix mutant model predicts an initial 5 to 10% of the lineages made of the original 2, which is not negligible. (TIFF) [file pgen.1009314.s018.tiff]

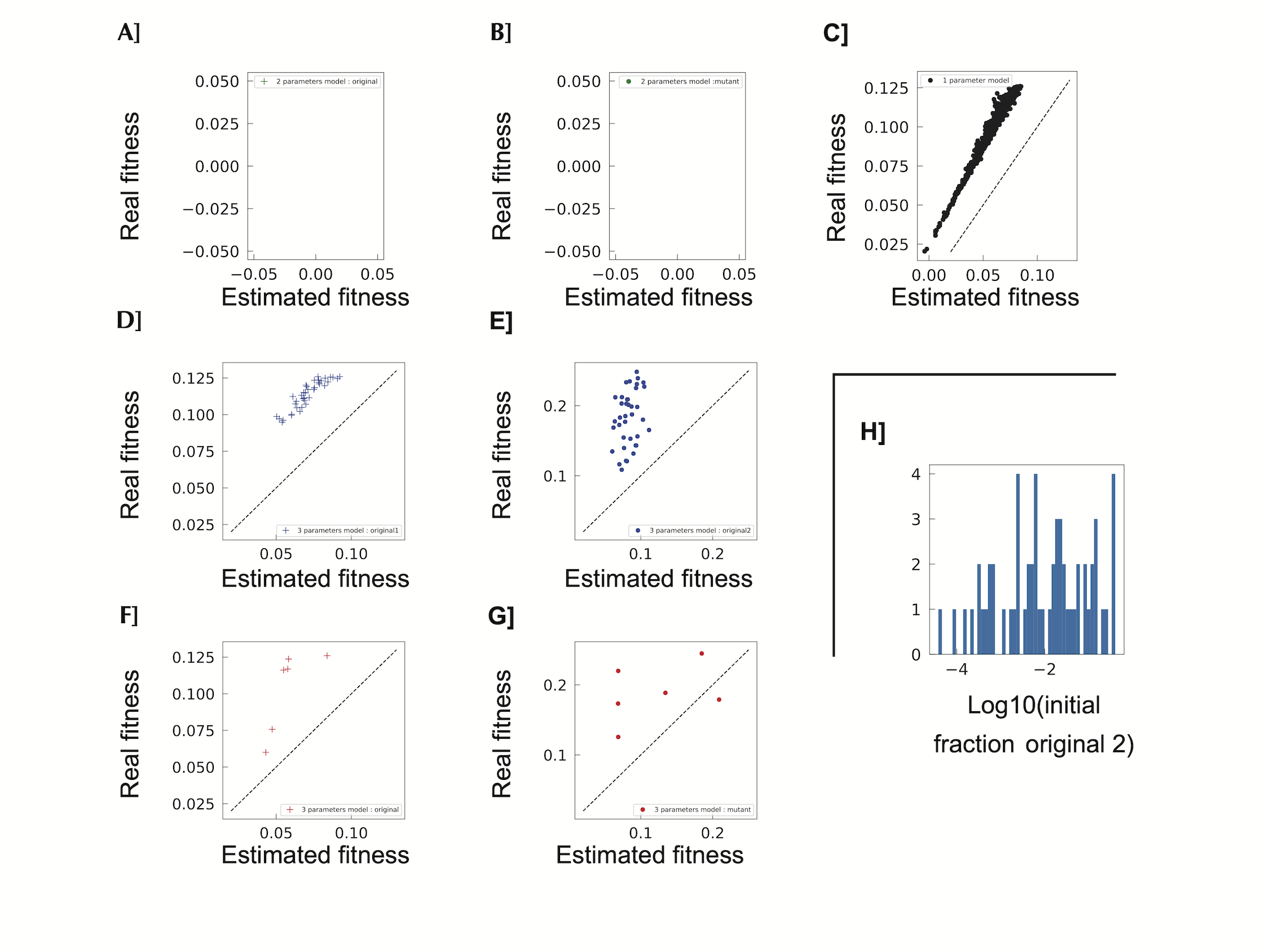

Supplement: S17 Fig — A] 2 parameters model: Original. B] 2 parameters model: Mutant. C] One parameter model. D] 3 parameters model initial mix of mutants: original 1 (larger fraction). E] 3 parameters model initial mix of mutants: original 2 (smaller fraction). F] 3 parameters model rising mutant: original. G] 3 parameters rising mutant: mutant. H] Distribution of fraction for the original 2 from 3 parameters model initial mix of mutants. (TIFF) [file pgen.1009314.s019.tiff]

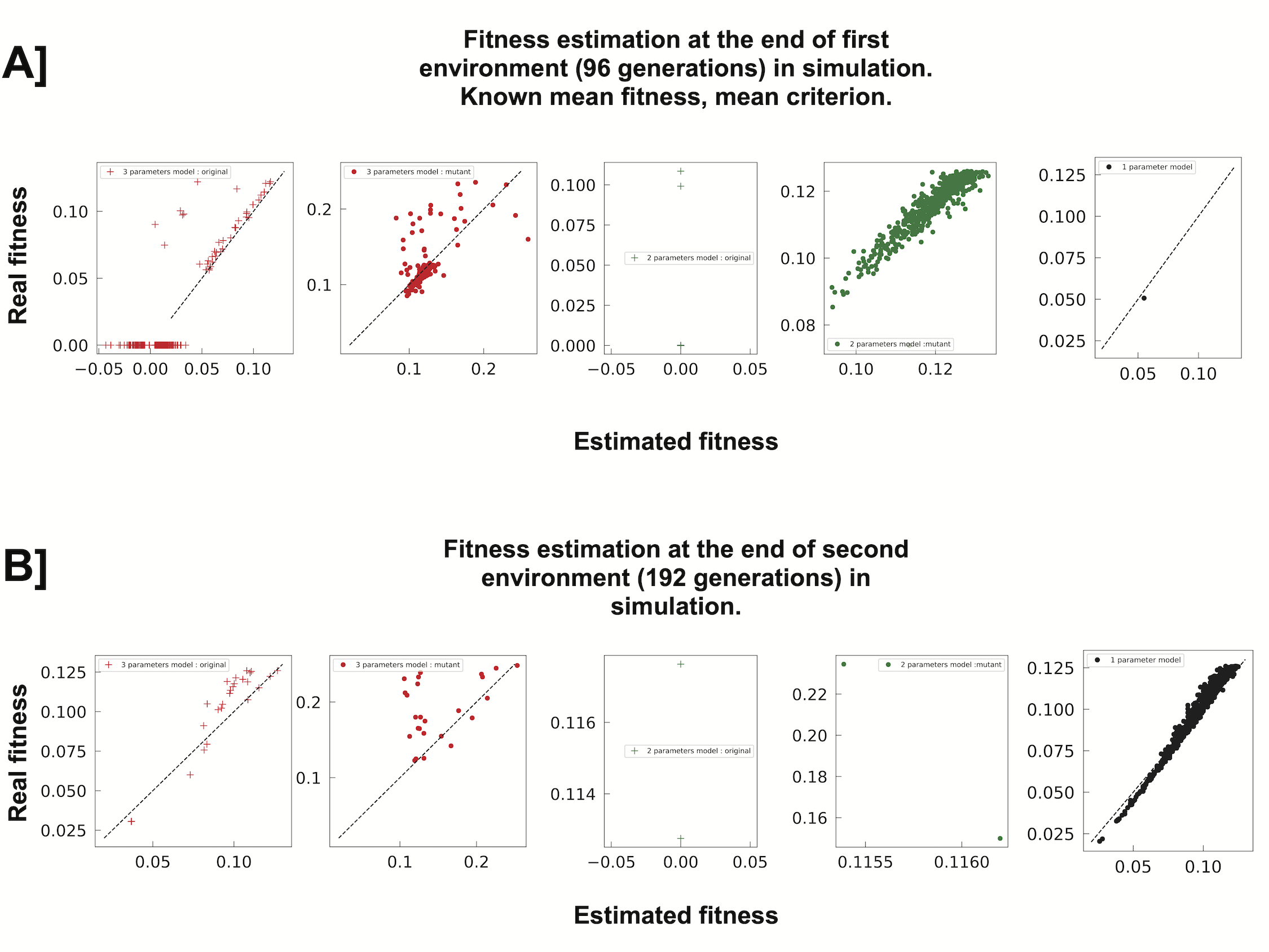

Supplement: S18 Fig — (TIFF) [file pgen.1009314.s020.tiff]

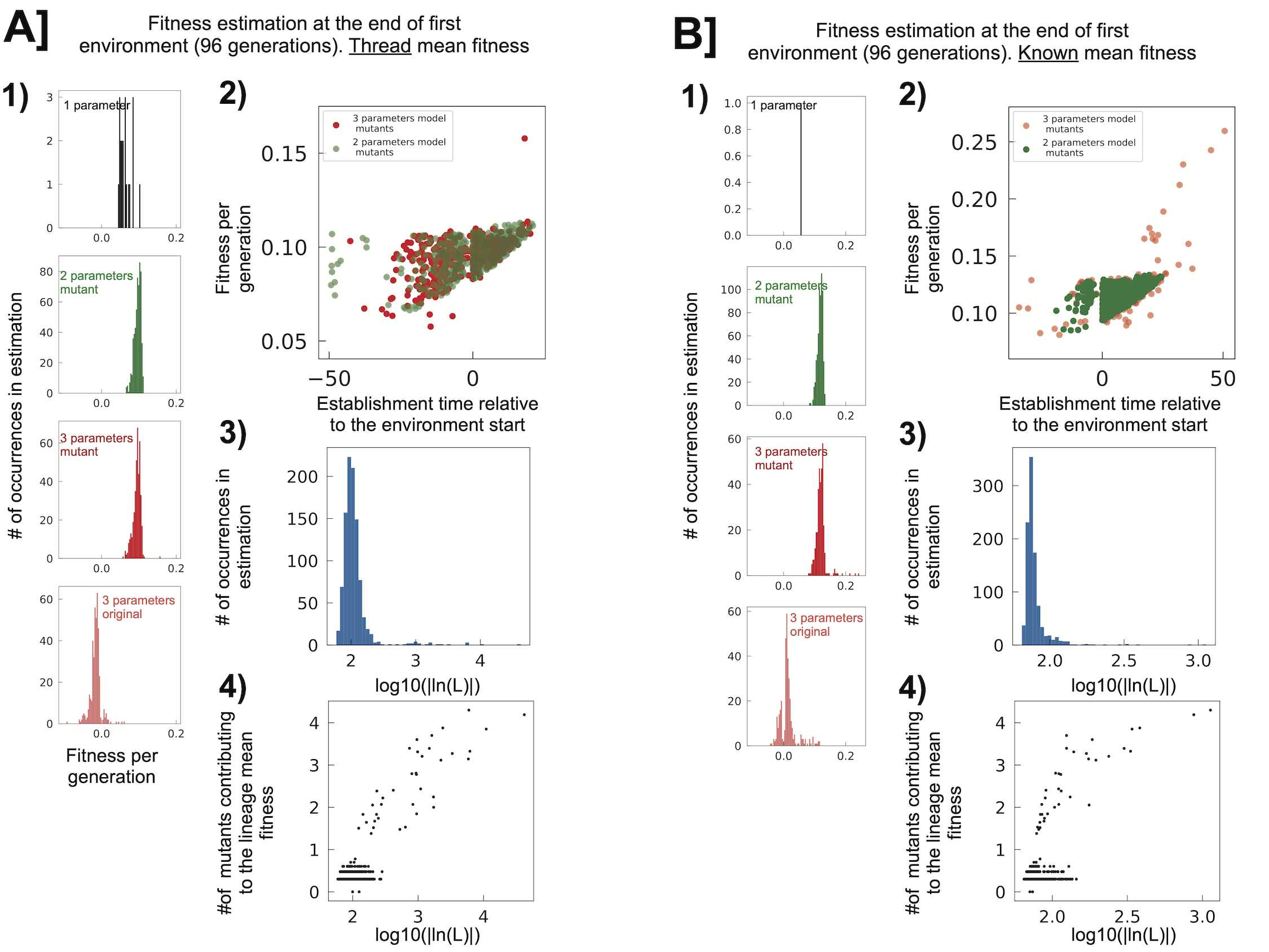

Supplement: S19 Fig — Overall comparison of the fits in environment 1 of simulation, of the first 1,000 largest lineages at the end of environment 1: A] Using for mean population fitness estimation either the thread method or B] Known lineages’ fitness. Using the population mean fitness calculated from known lineage fitness strongly increases the goodness of fit (comparing panels A]3) and B]3)). In terms of distribution of the different parameters there is no striking differences even though the two ways of calculating mean population fitness show sensible differences. (TIFF) [file pgen.1009314.s021.tiff]

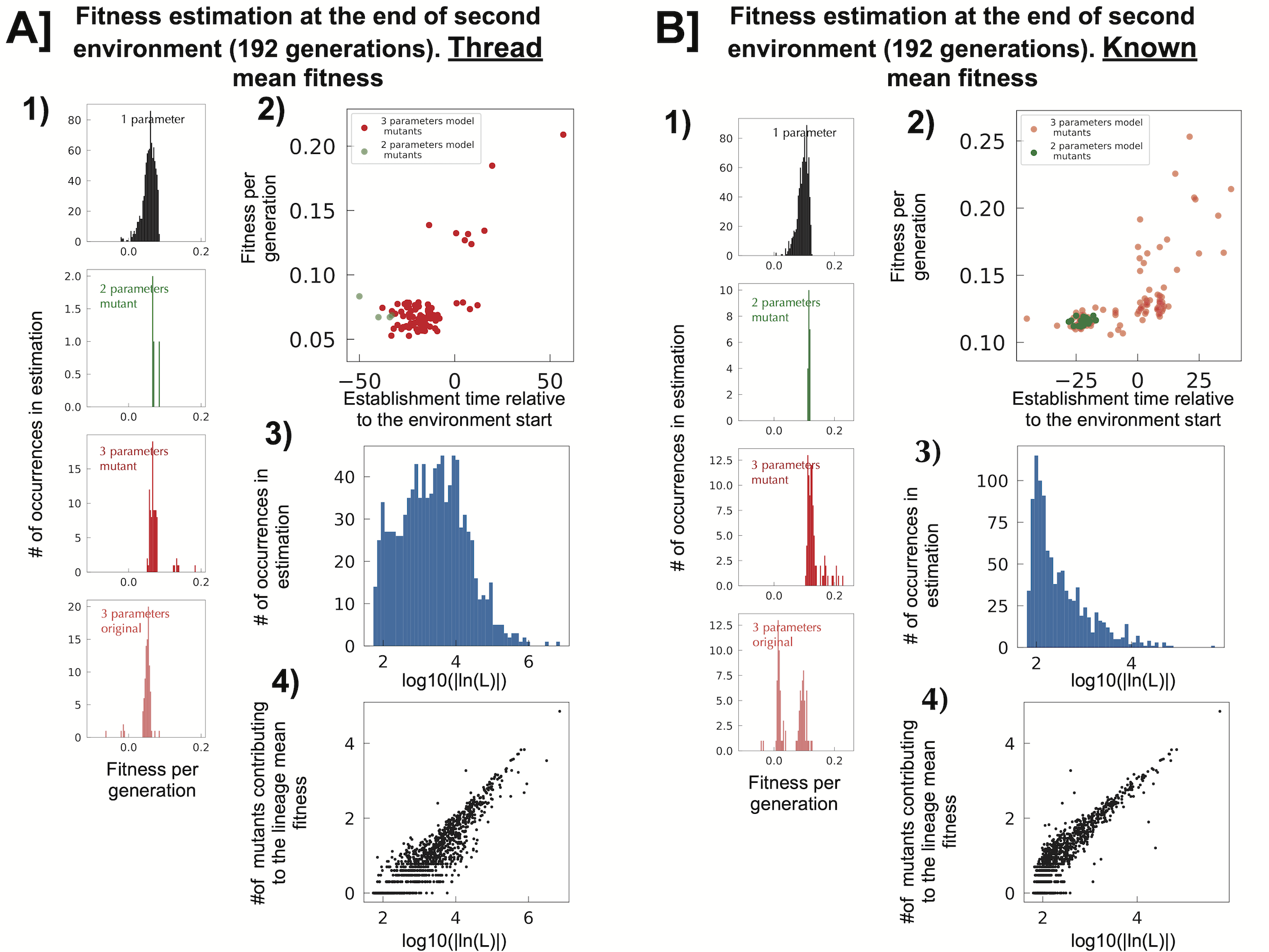

Supplement: S20 Fig — Overall comparison of the fits in environment 2 of simulation, of the first 1,000 biggest lineages at the end of environment 2: A] Using the thread method or B] Known lineages fitness for estimation of the population mean fitness. Using the mean fitness from known lineage fitness strongly increase the goodness of fit (comparing panels A]3) and B]3)). In terms of distribution of the different parameters there is no striking differences even though the two ways of calculating mean population fitness shows sensible difference (S12 Fig). (TIFF) [file pgen.1009314.s022.tiff]

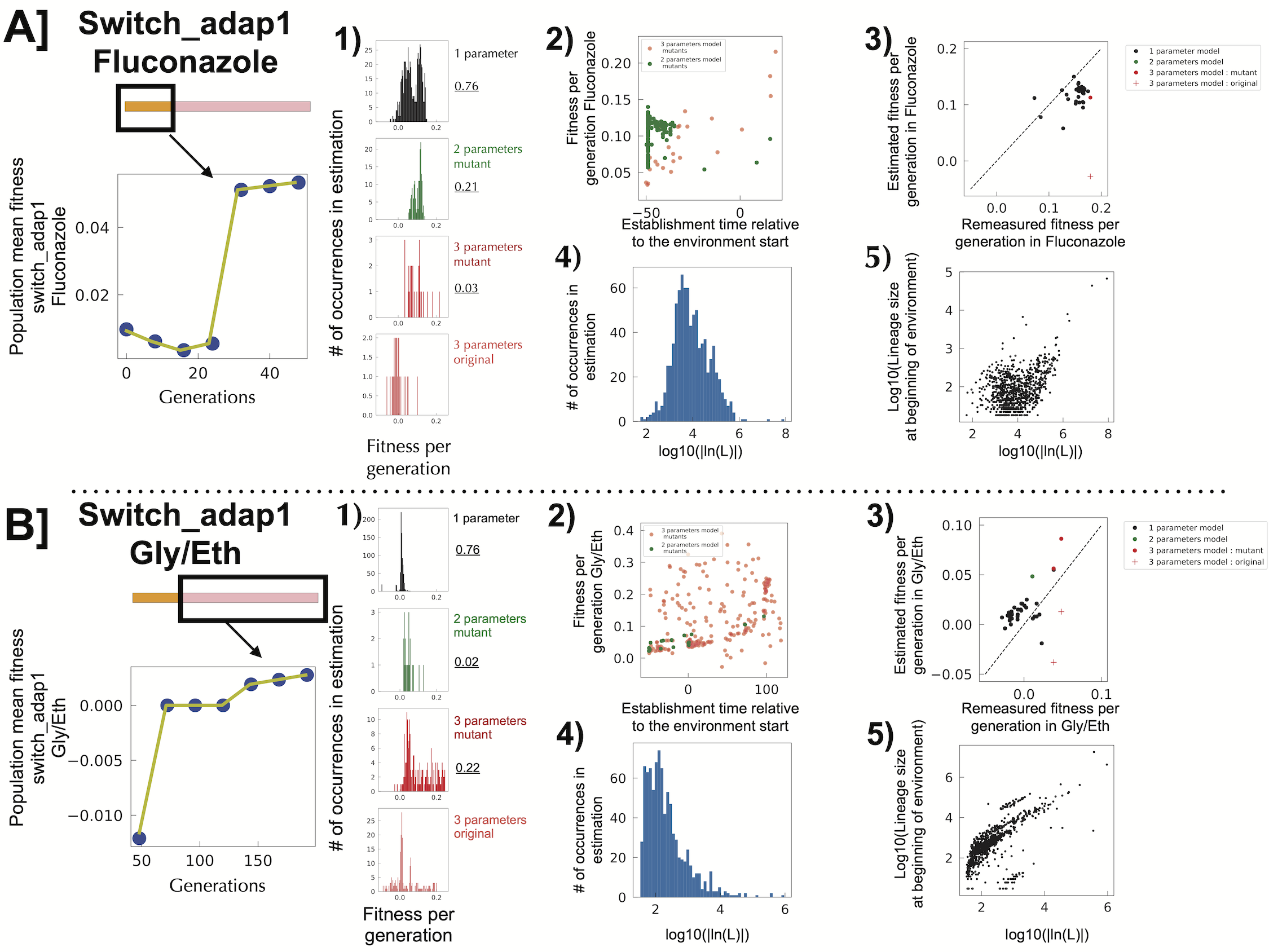

Supplement: S21 Fig — A] Analysis of the top 1,000 largest lineages at the end of the first environment. The most left panel is an estimation of the evolution of the population mean fitness using the exponential decay of lineages behaving similarly (thread). The population mean fitness function has a big jump in the middle, which follows well the type of behavior that we see in the lineage tracking, but which is not a behavior expected for usual population mean fitness. 1) Distribution of fitness effects according to the different model picked. The underlined number on the right of each panel represents the fraction of this particular model chosen by the algorithm. 2) Space phase for evolution in the first environment. 3) Fitness comparison between measured fitness and Maximum Likelihood estimation of fitness for the first environment of switch_adap1. The estimation follows quite well the measured fitness with of course an offset that comes from our estimation of mean fitness. 4) Distribution of log-likelihood for picked models. The fits are not very good as they are peaked around -10^4, whereas the good fit usually peaked around -10^2 (see simulations). This is probably coming from the big jump in mean fitness that we cannot explain. 5) Relationship between the size of a lineage at the beginning of an environment (as a proxy for the number of mutants contributing to the mean fitness of the lineage) and the goodness of the fit for those lineages. L is the likelihood of the model. B] Analysis of the top 1,000 largest lineages at the end of the second environment. Everything is smoother and makes more sense for the second environment. Still the goodness of fit is quite bad and should be reestimated using population mean fitness estimated from known lineages. (TIFF) [file pgen.1009314.s023.tiff]

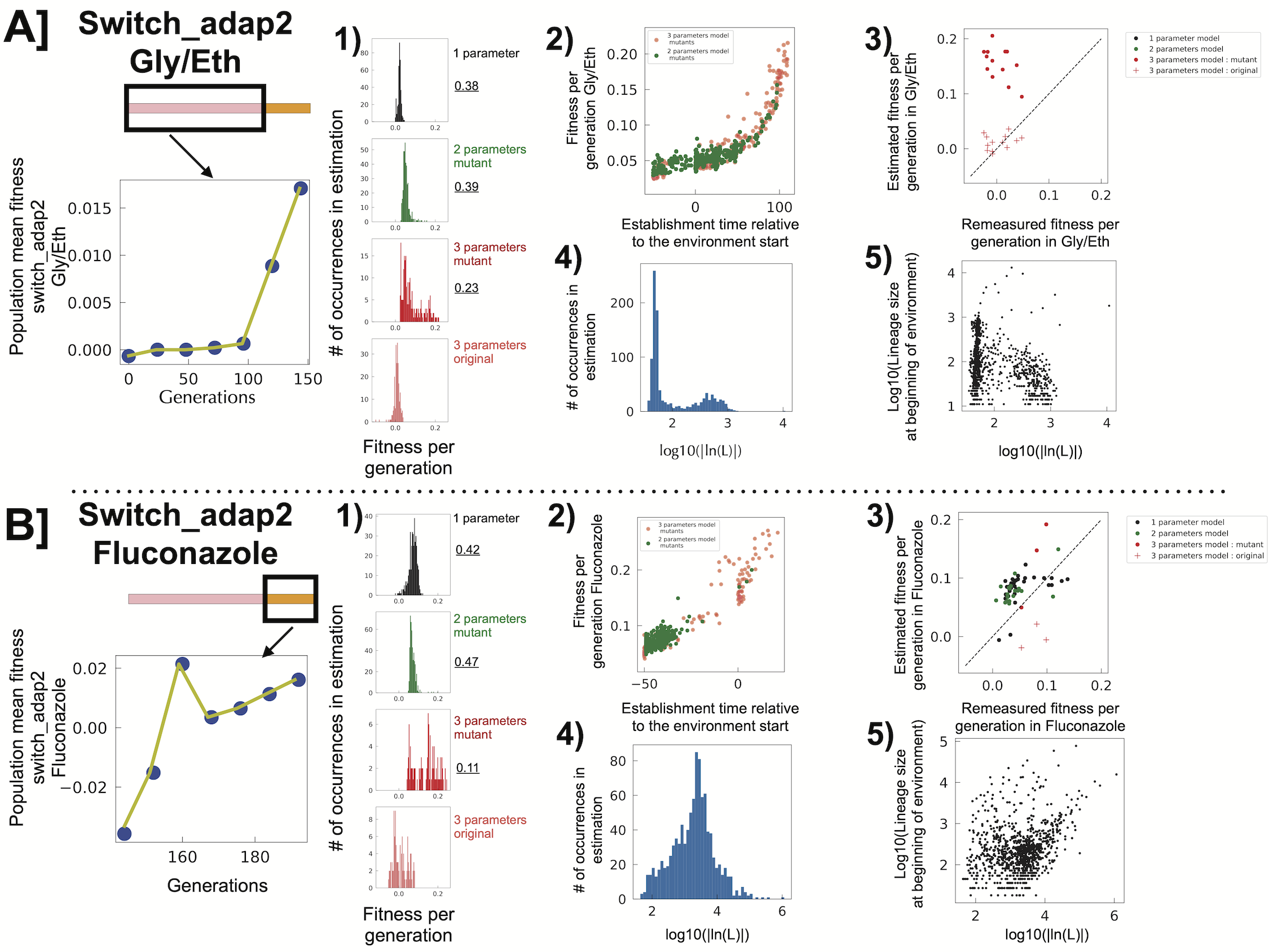

Supplement: S22 Fig — (TIFF) [file pgen.1009314.s024.tiff]

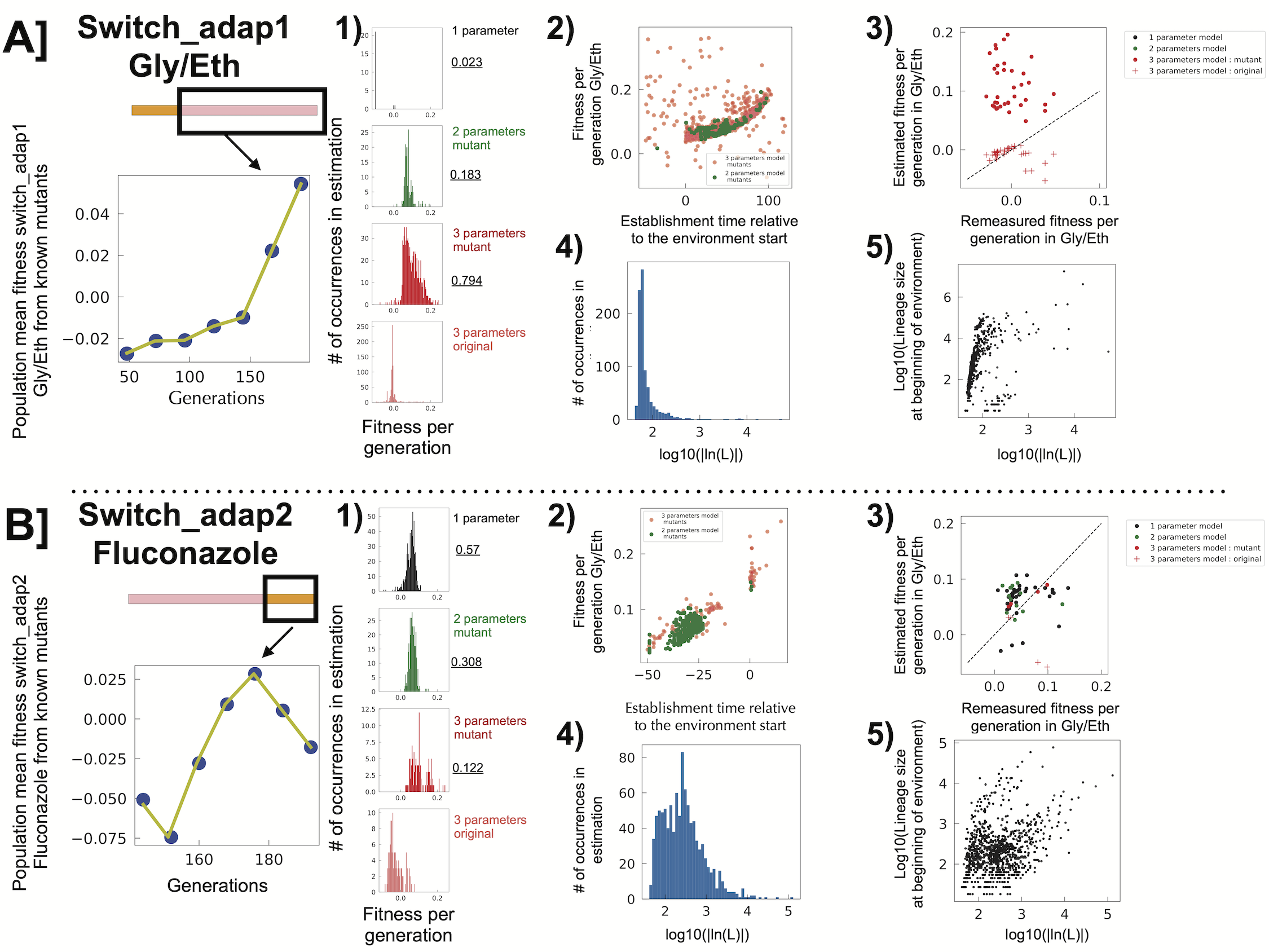

Supplement: S23 Fig — A] The goodness of fit distribution 3) is orders of magnitude better than with the thread way to calculate population mean fitness. In addition, most of the 1 parameter models seen before have been moved to a three parameters model. B] The goodness of fit distribution 3) is orders of magnitude better than before even though still being quite large. If one looks at the mean population function associated with that Fluconazole environment in all our experiments, it is obvious that we are lacking a full description of that environment. (TIFF) [file pgen.1009314.s025.tiff]

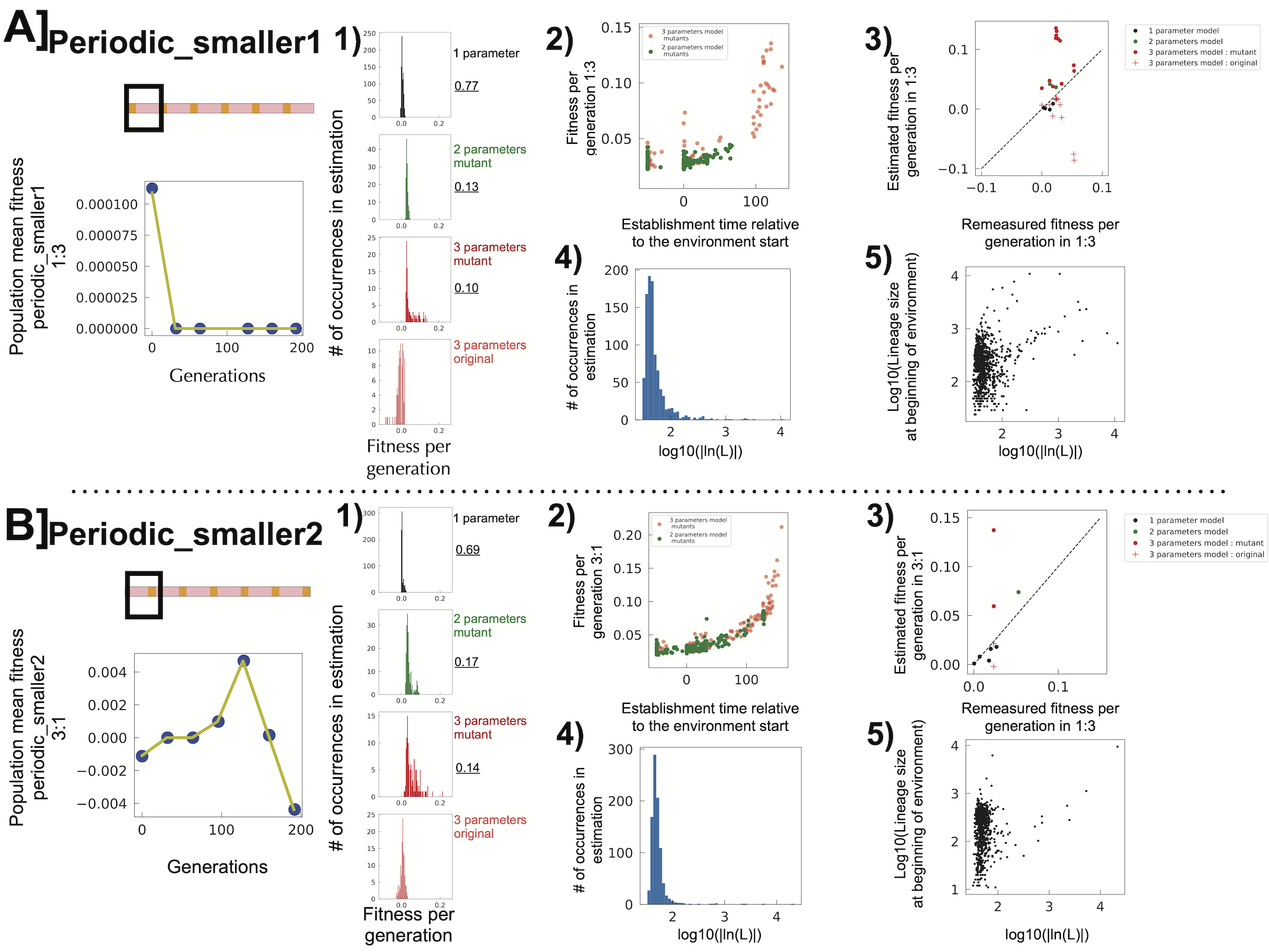

Supplement: S24 Fig — (TIFF) [file pgen.1009314.s026.tiff]

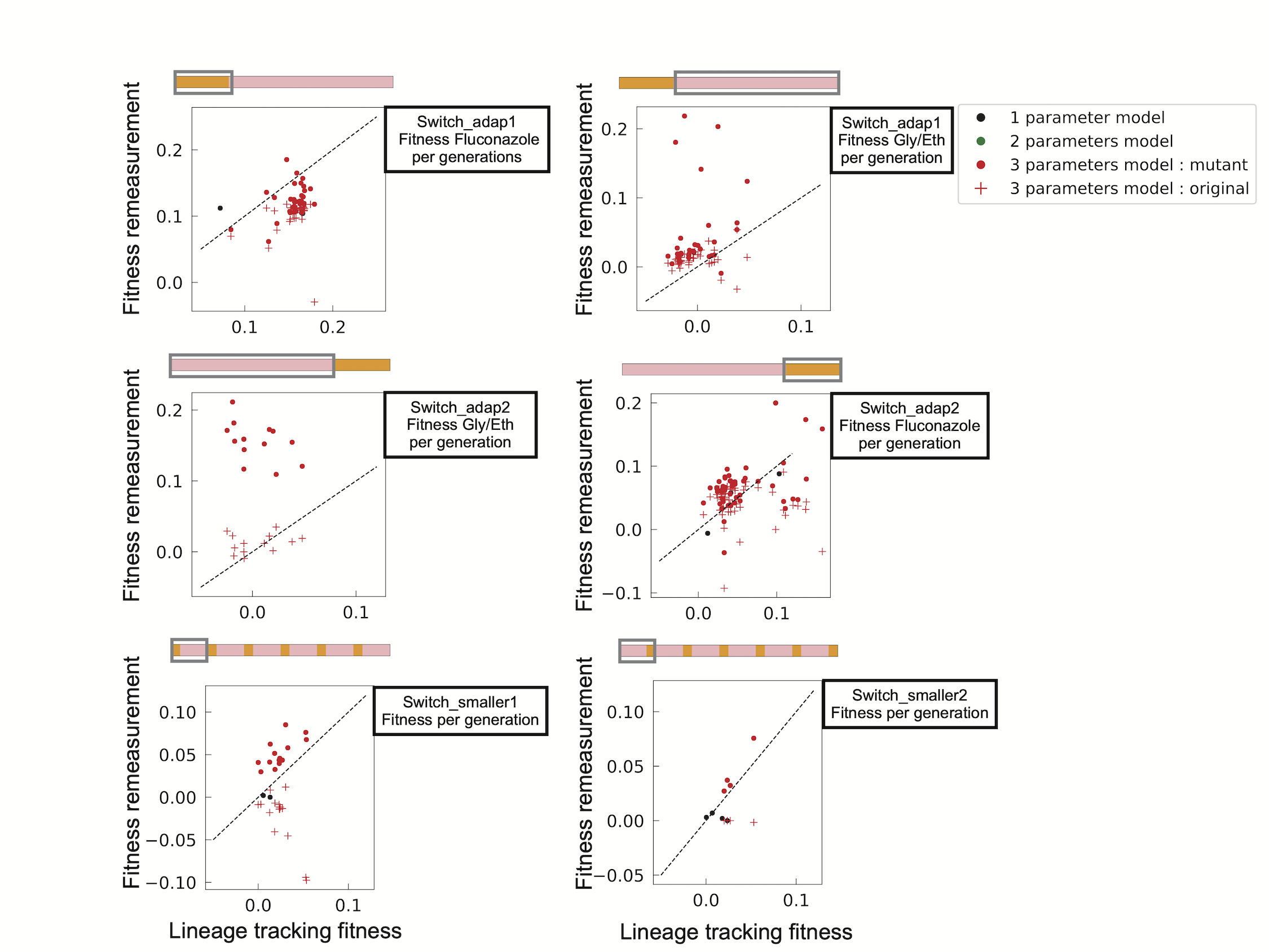

Supplement: S25 Fig — (TIFF) [file pgen.1009314.s027.tiff]
